# Occupational exposure and markers of genetic damage, systemic inflammation and lung function: a Danish cross-sectional study among air force personnel

**DOI:** 10.1038/s41598-021-97382-5

**Published:** 2021-09-09

**Authors:** Maria Helena Guerra Andersen, Anne Thoustrup Saber, Marie Frederiksen, Per Axel Clausen, Camilla Sandal Sejbaek, Caroline Hallas Hemmingsen, Niels E. Ebbehøj, Julia Catalán, Kukka Aimonen, Joonas Koivisto, Steffen Loft, Peter Møller, Ulla Vogel

**Affiliations:** 1 The National Research Centre for the Working Environment, Lersø Parkallé 105, 2100 Copenhagen Ø, Denmark; 2 Department of Occupational and Environmental Medicine, Bispebjerg University Hospital, Bispebjerg Bakke 23, 2400 Copenhagen, NV, Denmark; 3 Finnish Institute of Occupational Health, P.O. Box 40, 00032 Työterveyslaitos, Helsinki, Finland; 4 Department of Anatomy, Embryology and Genetics, University of Zaragoza, 50013 Zaragoza, Spain; 5 ARCHE Consulting, Liefkensstraat 35D, 9032 Wondelgem, Belgium; 6 Department of Public Health, Section of Environmental Health, University of Copenhagen, Øster Farimagsgade 5A, 1014 Copenhagen K, Denmark; 7 Department of Health Technology, Technical University of Denmark, 2800 Kgs, Lyngby, Denmark

**Keywords:** Biomarkers, Risk factors, Environmental chemistry

## Abstract

Air force ground crew personnel are potentially exposed to fuels and lubricants, as raw materials, vapours and combustion exhaust emissions, during operation and maintenance of aircrafts. This study investigated exposure levels and biomarkers of effects for employees at a Danish air force military base. We enrolled self-reported healthy and non-smoking employees (n = 79) and grouped them by exposure based on job function, considered to be potentially exposed (aircraft engineers, crew chiefs, fuel operators and munition specialists) or as reference group with minimal occupational exposure (avionics and office workers). We measured exposure levels to polycyclic aromatic hydrocarbons (PAHs) and organophosphate esters (OPEs) by silicone bands and skin wipes (PAHs only) as well as urinary excretion of PAH metabolites (OH-PAHs). Additionally, we assessed exposure levels of ultrafine particles (UFPs) in the breathing zone for specific job functions. As biomarkers of effect, we assessed lung function, plasma levels of acute phase inflammatory markers, and genetic damage levels in peripheral blood cells. Exposure levels of total PAHs, OPEs and OH-PAHs did not differ between exposure groups or job functions, with low correlations between PAHs in different matrices. Among the measured job functions, the UFP levels were higher for the crew chiefs. The exposure level of the PAH fluorene was significantly higher for the exposed group than the reference group (15.9 ± 23.7 ng/g per 24 h vs 5.28 ± 7.87 ng/g per 24 h, p = 0.007), as was the OPE triphenyl phosphate (305 ± 606 vs 19.7 ± 33.8 ng/g per 24 h, p = 0.011). The OPE tris(1,3-dichlor-2-propyl)phosphate had a higher mean in the exposed group (60.7 ± 135 ng/g per 24 h) compared to the reference group (8.89 ± 15.7 ng/g per 24 h) but did not reach significance. No evidence of effects for biomarkers of systemic inflammation, genetic damage or lung function was found. Overall, our biomonitoring study show limited evidence of occupational exposure of air force ground crew personnel to UFPs, PAHs and OPEs. Furthermore, the OH-PAHs and the assessed biomarkers of early biological effects did not differ between exposed and reference groups.

## Introduction

Air Force ground crew personnel perform diverse tasks such as aircraft inspection and maintenance, aircraft runway operations, tank fuel or munitions installation and inspection. This can result in exposure to fuels and lubricants as raw materials, vapours and exhaust emissions, potentially containing complex mixtures of chemicals. Jet propellant fuel 8 (JP-8) is produced according to a stringent internationally agreed standard used by North Atlantic Treaty Organization (NATO). It is a kerosene fuel with performance additives for military use. Kerosene-based fuels consist of a complex mixture of aliphatic and aromatic hydrocarbons including potential carcinogenic compounds such as benzene, ethylbenzene and naphthalene (which together make up ≤ 1% volume/volume)^1^. Interactions among the constituents and additives are unknown, and earlier reports have highlighted that JP-8 has inconclusive toxicological effects^1,2^. The repeated nature of occupational exposure to raw fuel, vapours and exhaust, raises concerns relating health effects to the immune system, respiratory tract, central nervous system and genotoxicity^3^. Another concern relates to the extensive use of organophosphate esters (OPEs) in aircraft lubricating oils and hydraulic fluids, in addition to their use as flame retardants and plasticizers^4^. Concentrations of OPEs in air, soil and pine needles have been found to decrease with increasing distance from an airport, suggesting dispersion of jet fuel fumes in the local environment^5^. OPE metabolites have frequently been detected in human urine but occupational exposure studies are scarce^6^. Toxicological studies focusing on OPEs have reported evidence of endocrine, reproductive, neurological and systemic effects, and for chlorine-containing OPEs potential carcinogenic effects^7^.

We have previously measured ultrafine particle (UFP) levels in connection with personnel assisting the take-off and reception of aircrafts inside a hangar at a non-commercial airfield. Both measured particle peak levels and animal studies performed with particulate material collected on site raised concerns about the occupational exposure and eventual health effects thereof^8^.

In the present study, we aimed to investigate the occupational exposure to UFPs, polycyclic aromatic hydrocarbons (PAHs) and OPEs and markers of biological effects among ground crew personnel at a military air base in Denmark.

## Materials and methods

### Study design and study participants

We recruited employees at a Danish Air Force base for exposure and health effects characterization, using a cross-sectional design. Self-reported smoking, pregnancy, and drug or alcohol misuse were exclusion criteria. Among approximately 700 employees at the base, we enrolled 79 persons, 42 of whom had job functions with potential exposure through dermal and/or inhalation routes to fuel vapours, lubricants and jet exhaust, including crew chiefs (n = 17), aircraft engineers (n = 14), fuel operators (n = 6) and munition specialists (n = 5). We defined the reference group as 37 other military staff presently working as office workers (n = 31) or avionics (n = 6) at the base. The study was conducted in May and June 2018, with all data and biological samples collected in four campaigns. The biological material was always collected on Thursdays. The Danish Air Force reported similar air traffic in terms of departures and arrivals of airplanes. The sample collection was stratified into campaigns in order to increase a possible exposure gradient (i.e. considering variability in flight and maintenance activities) and limitations in processing of biological samples, whereas the whole period was kept short to avoid period effects in the biomarkers due to systematic changes of independent risk factors. Data collection included UFP breathing zone measurements, skin wipes, silicone bands, urine and blood samples, lung function measurements and questionnaires filled in on the campaign day. The Danish Committee on Health Research Ethics of the Capital Region approved the study (H-17029207). All study participants received both oral and written information and provided written consent before enrolment. All methods were carried out in accordance with relevant guidelines and regulations.

### Assessment of information from participants

Self-reported information on participants characteristics concerning anthropometric data, working history, lifestyle factors, use of personal protective equipment (PPE), health history and medication intake was assessed through questionnaires which were filled in at the end of the working shift.

### Measurement of personal exposure to ultrafine particles

UFP exposure was measured in the breathing zone of the employee with a portable diffusion charger device (DiSCmini, Matter Aerosol AG, Wholen, Switzerland). We used 4 devices per sampling day and they were randomly distributed among those enrolled participants in different job functions and locations, who were willing to carry the devices. The final distribution between job functions was: crew chiefs (n = 2), aircraft engineers (n = 2), munition specialists (n = 2) and office workers (n = 7). Due to logistic issues, the data collection was limited to four working hours in the morning shift. We used the recording resolution of 1 s in the data analyses.

### Exposure sample collection on skin wipes

We assessed PAHs on skin wipes. A research staff member sampled the skin wipes on the campaign days. Three sampling sites on the neck and both hand palms were wiped with an alcohol wetted wipe for each sample (70% isopropanol/water, Mediq Denmark A/S) as previously described^9^. Briefly, on the campaign day each of the skin areas were wiped twice with the same wipe, first with one side of the wipe and then with the other side. The research staff used nitrile gloves, which were changed between subjects. The wipes were placed in glass vials, kept in the dark and transported to the laboratory on the same day. The wipe samples were stored at -18℃ until extraction and analysis. Extracts of four field blank wipes kept in glass vials during sampling were analysed in parallel with each series of wipe samples. A nominal area of 18 cm^2^ for the neck wipes and 160 cm^2^ for each hand palm were used in the calculations.

### Exposure sample collection on silicone bands

We used silicone bands (202 × 12 × 2 mm, Nordic Wristbands, Denmark) to assess exposure to PAHs and OPEs (Table S1). Prior to use, the silicone bands were pre-cleaned at 280 °C for at least 5 h and stored at room temperature in sealed Rilsan bags. They were distributed to each study participant with instructions to wear them during working hours from Monday to Wednesday as a pendant on their clothes. The silicone bands were returned to the research staff on the campaign day, on Thursdays. The participants were instructed to store it in a Rilsan bag in the dark when off-duty. They also received instructions to log the use of the silicone band (time of start and end for each day). For each campaign round, one field-blank silicone band was analysed in parallel.

### Urine and blood sample collection

On the campaign day, each study participant delivered a first morning spot urine sample, which was transported to the laboratory and kept at -20℃ until analysis. Peripheral venous blood samples were collected in vacutainer heparin-coated tubes for whole blood, ethylenediaminetetraacetic acid (EDTA)-coated tubes for plasma preparation, and vacutainer cell preparation tubes for isolation of peripheral blood mononuclear cells (PBMCs) (Vacutainer® Becton Dickinson A/S, Brøndby, Denmark). The plasma and PBMC samples were prepared in an office room at the military base (with centrifuge Hettich, Universal 16, Bie&Berntsen A/S, Denmark), kept on ice and transported on ice to the laboratory in the same day. Plasma was prepared by 10 min centrifugation at 1780 g. PBMCs were separated by 20 min centrifugation at 1140 g. The PBMCs were diluted with 3 mL ice-cold medium (Rosswell Park Memorial Institute, RPMI, medium with 10% foetal bovine serum and 1% Pen/Strep) and kept on ice. At arrival to the laboratory, the PBMCs were pelleted by 15 min centrifugation at 300 g at 5 °C (centrifuge Sorvall, RC-6, Axeb A/S, Denmark) and re-suspended in 3 mL RPMI medium with 10% foetal bovine serum and 1% Pen/Strep. The same centrifugation procedure was repeated, and the PBMCs were re-suspended in freezing medium (RPMI with 50% foetal bovine serum and 10% DMSO). The plasma and PBMCs were stored at − 80 °C until analysis, and the whole blood samples were kept at 4 °C until expedition for analysis.

### Lung function measurements

Lung function was assessed with the Easy on-PC Spirometer (ndd, Medical technologies, Zurich, Switzerland), measuring forced vital capacity (FVC), forced expiratory volume in one second (FEV1) and peak expiratory flow (PEF), besides the determined percentage of predicted values, accordingly with device settings for ethnicity of the measured subjects. The participants received instructions and performed the test standing upright. At least three acceptable manoeuvres were performed to obtain reproducible tracings. Data acquisition quality was checked with all results acceptable for analysis.

### Analysis of PAHs from skin wipes

The extraction and analysis of PAHs were performed as previously described in Andersen et al., 2018^9^. Briefly, the wipes were treated with cyclohexane, sonicated for 30 min in an ultra-sonic bath (Branson 5200, output power 120 W at extraction of 25 samples at the same time). One millilitre of supernatant was transferred to a glass vial and added 30 µL of internal standard solution (10 ng/µL). The extracts were stored at − 18 °C until analysis. We analysed the extracts by gas chromatography and mass spectrometry (GC–MS) using a Brucker SCION TQ (Bruker Daltonics, Bremen, Germany). The analysis was performed by injection of 1 µL of the sample extract with an auto-sampler to a programmable temperature vaporising injector at 280 °C into the column (VF-5MS, Agilent Technologies, USA) with helium flow of 1 mL/min. The GC oven programme was set at 70 °C for 4 min, ramp 1, 10 °C /min to 300 °C, ramp 2, 45 °C /min to 325 °C. The MS was operated in a scan mode in electron ionization and in selected ion monitoring for each PAH. Field blanks from each campaign were run alongside, some analytes were detected in very low levels and all samples were blank corrected. The results of the sum of the 3 wipes for each study participant were normalized to mass per hour to correct for the different sampling timings because the wiping was done 1–4 h after check-in in morning shift function work.

### Analysis of PAHs and OPEs from silicone bands

Approximately half of the silicone band (2.5 g) was used for analysis. It was cut into smaller pieces, weighted and transferred to 5 mL ASE-cells and isotope-labelled internal standards were added. The extractions were carried out by pressurized liquid extraction (Dionex, ASE-300) with *n*-hexane:acetone (1:1), static time 10 min at 125 °C, 4 cycles, 60% flush and 60 s. purge. The extracts were concentrated to approximately 1 mL using a Genevac Rocket Synergy Evaporator (Thermo Fisher Scientific) using isooctane as keeper. The extracts were analysed for PAHs and OPEs by GC–MS–MS using the GC-programme described for PAHs on wipes; ions for OPEs were added to the method. Field blanks from each campaign were run alongside, some analytes were detected in very low levels and all samples were blank corrected. The results were expressed in ng per g of silicone band and were normalized to 24 h to account for different deployment times among participants, as some had absent days among in addition to the daily variation of working hours.

### Analysis of PAH metabolites in urine

We measured levels of the urinary PAH metabolites 1-hydroxynapthalene (1-NAP), 2-hydroxynapthalene (2-NAP), 2-Hydroxyfluorene (2-FLU), 1-hydroxyphenanthrene (1-PHE), 2 and 3-hydroxyphenanthrene (2 + 3-PHE), 4-hydroxyphenanthrene (4-PHE) and 1-hydroxypyrene (1-PYR). The analysis followed the method previously described^10^ with minor changes. The sample volume was increased to 3 mL, native and ^13^C-labelled internal standards were obtained from Cambridge Isotope Laboratories (Andover, MA, USA) and hydroxy fluorene and phenanthrenes were added to the method. The eluate was evaporated under reduced pressure using a Genevac Rocket Synergy Evaporator. The remaining residue was re-dissolved in 100 µL methanol. Each batch included 14 samples/calibration including as well one blank and one reference material sample. Synthetic urine (Surine™ Negative Control Urine) was used for calibration standards and procedural blanks, no analytes were detected above limit of quantification (LOQ) in the blanks. Reference material samples were prepared in the same manner using NIST SRM 3672 smokers’ urine. The results of the reference material were within 70–106% of the certified values with relative standard deviations of < 10%, except for the hydroxy-phenanthrenes, where interferences resulted in greater uncertainties. All urine concentrations were standardized for diuresis with the concentration of urinary creatinine, as previously described^11^.

### Analysis of inflammation markers

We determined serum amyloid A (SAA) and C-reactive protein (CRP) in plasma by enzyme-linked immunosorbent assay (ELISA) kits from Invitrogen (CA, USA) and IBL International GMBH (Hamburg, Germany), respectively, as previously described^12^.

### Analysis of DNA damage

DNA damage was determined by the comet assay on the isolated PBMCs, quantified as percentage of DNA in tail (%DNA) and tail length (TL), scored by PathFinder™ system (IMSTAR, Paris, France), as described previously^13^. Briefly, the cells were embedded in agarose (0.70% final concentration of low melting point agarose with phosphate-buffered saline (PBS)) and cell suspensions deposited on CometSlide™ 20-well (Trevigen, MD, USA), then immersed in cold lysing solution (2.5 M NaCl, 10 mM Tris-Base, 100 mM Na_2_EDTA, 1% Na-sarcosinate, 10% DMSO, 1% Triton X-100, pH 10) and kept overnight at 4 °C. Subsequently, the samples were alkaline treated for 40 min and subjected to electrophoresis (38 V, 0.7–0.8 A, 1.15 V/cm, from anode to cathode, for 25 min) under cold (4 °C) conditions. All samples were included in the same electrophoresis run, disposed in 5 slides. Thereafter, slides were neutralized in neutralization buffer (0.4 M Tris, pH 7.5), fixed with 96% ethanol, stained with SYBRGreen® and scored by the fully automated PathFinder™ system (average of 3163 objects analysed). As assay controls, we used A549 cells (human lung epithelial cell line) treated with H_2_O_2_ (45 µM, for 30 min at 4 °C) or PBS, both controls added to all 5 slides (assay control results in Table S2). The primary comet assay endpoint (%DNA in the comet tail) was transformed to lesions per 10^6^ base pairs (bp) using the conversion factor of 0.036574 (from the calibration curve in supplementary Figure S1), assuming the well-established relationship between ionizing radiation dose and yield of strand breaks in DNA, applying the previously described algorithm^14^.

### Analysis of micronuclei frequency

Chromosome damage was assessed in transferrin-positive peripheral blood reticulocytes by the micronucleus assay using flow cytometry, as described elsewhere^15^. Briefly, the whole blood was processed within 4 days after collection. Immunomagnetic separation of transferrin-positive (+CD71) reticulocytes was performed according to the instructions of the CELLection™ Pan Mouse IgG Kit (Invitrogen, Thermo Fisher Scientific, MA, USA) using a FITC Mouse Anti-human CD71 antibody (BD Biosciences, CA, USA). Isolated + CD71 reticulocyte samples were fixed in 2% paraformaldehyde in PBS with 10 µg/ml of sodium dodecyl sulfate (SDS; Sigma-Aldrich, Merck KGaA, Darmstadt, Germany) and kept refrigerated (4 °C) until flow cytometric analysis. Prior to the analysis DNA was stained with Hoechst 33,342 (Invitrogen, Thermo Fisher Scientific, MA, USA). Samples were analyzed with CytoFlex S-flow cytometer (Beckman Coulter, IN, USA) using blue (488 nm) laser for the identification of + CD71 reticulocytes and near UV (375 nm) laser for the detection of DNA-containing micronuclei. Data was collected and analyzed with Beckman Coulter CytExpert Acquisition and Analysis software version 2.3. The micronuclei frequency was quantified as per-mille of micronucleated + CD71 reticulocytes from all analysed + CD71 reticulocytes. A minimum of 20 000 + CD71 reticulocytes per sample were required to ensure reliable data, resulting in the exclusion of 9 samples. The number of analysed + CD71 reticulocytes was not different among the groups and is presented in supplementary Table S3.

### Statistics

The results were analysed using the statistical software R version 3.5.3. For questionnaire data, categorical variables were summarized using counts and frequencies and p values obtained using the function *utable* from the package *Publish*^16^. PAHs, OPEs and OH-PAHs were not detected in all samples (Table S1), therefore the levels < LOQ were imputed based on three different approaches, i.e. substituting with zero, 1/2LOQ and the “best estimates”, which is the concentration estimated disregarding the official LOQ. In the statistical analysis we used the best estimates to avoid either many zeros or artificially high exposure levels. Furthermore, correlation analyses were performed only including compounds with detection frequencies higher than 70% in order to minimize the impact bias of imputed data. Because of skewed distribution of data, total PAHs, OH-PAHs, CRP, SAA, and micronuclei frequency were logarithmically transformed and OPEs and DNA strand breaks were cubic root transformed (details in supplementary Table S4). The Welch one-way test was used to compare group means accounting for the groups different sample sizes (with the function *oneway.test*) and for the analysis of individual OPEs, the non-parametric Wilcoxon rank sum test was used (with the function *wilcon.test*). The Hotelling T-squared test was used to compare the combined means for related outcomes: inflammation (CRP and SAA), genetic damage (DNA strand breaks and micronuclei frequency) and lung function (FVC, FEV1 and PEF) in a multivariate analysis of means for the two groups (using the function *hotelling.test*). Assumptions were checked using the function *mshapiro.test* from *mvnormtest* package and the functions *cov* and *det* (for the determinant of variance–covariance matrix for the variables combinations). Female participants were excluded in a sensitivity analysis. Additionally, one complementary analysis was also made excluding participants with potential exposure (avionics and four office workers with office located in the hangar) from the reference group. The linear model was fitted with the function *lm* where the response was the output variable of interest with a series of terms to specify a linear predictor. The terms were the exposure category adjusted for confounders, i.e. age, sex, BMI, relevant health history and for lung function also smoking history (confounders chosen from directed acyclic graph (DAG) presented on supplementary Figures S2, S3 and S4). The relevant health history considered for lung function was self-reported diagnose of asthma; for inflammation it was diabetes and eczema; and for genetic damage it was diabetes, stroke and cancer^17^. The campaign day is not included in the statistical models, because it was the same day of the week (Thursday) and the four campaigns are considered to be linked to the exposure status, whereas the biomarkers are not expected to be affected by period effects such as seasonal variation in the relatively short sample collection period. For the linear regression, the normality assumption was checked for the residuals of the model, and because of skewness the variables micronuclei frequency, CRP and SAA were logarithmically transformed.

## Results

### Demographic characteristics

Table 1 shows the self-reported characteristics of the study participants per group (reference and exposure groups, as defined in study design). The age of all participants ranged from 25 to 61 years with an average age of 46.9 years (± 10.3 years) and with a majority of males (87%). This is in line with the air base working force composition with an average age of 44 years and 91% males. In terms of body mass index (BMI), 32% of the participants had between 21.5–24.9 kg/m^2^, 54% between 25–29.9 kg/m^2^, and 14% between 30–37.6 kg/m^2^. The defined exposed group was not significantly different from the reference group with regards to the majority of the characteristics presented. Two exceptions were medication intake and the years of work in the exposure areas (hangar and operative area), with the last expected to be different. A total of 31 participants reported to use at least one type of medication in the last 14 days (excluding nutrient supplements), which accounted for 58% (n = 21) of the reference participants and 26% (n = 10) of the exposed participants. Although none of the participants from either group reported having a history of cardiovascular disease, 9 reported intake of prescribed medication for high blood pressure, which was probably not perceived as a cardiovascular condition. Four participants reported to have extra activities with potential particle exposures. Self-reported information about the use of PPE is presented in supplementary Table S5. The self-reported use of PPE showed that the majority of the employees in the exposed group used gloves when performing tasks entailing skin exposure, whereas inhalation protection equipment was less used.

**Table 1:** Self-reported characteristics of the study participants.

Characteristic	Exposed group (n = 42)	Reference group (n = 37)	P value
Age (years)	46 (± 11.8)	48 (± 8.5)	0.398
Sex (male)	40 (95%)	29 (78%)	0.056
Height (cm)	180.6 (± 8.4)	177.9 (± 9.4)	0.185
Weight (kg)	88 (± 14.4)	86.8 (± 13.8)	0.697
BMI^a)^ (kg/m^2^)	26.9 (± 3.4)	27.3 (± 3.3)	0.590
Normal (18.5–24.9)	15 (36%)	10 (27%)	
Overweight (25.0–29.9)	20 (48%)	23 (62%)	
Obese (≥ 30)	7 (17%)	4 (11%)	
Employment duration in the air base			0.099
< 1 year	0	0	
1–10 years	18 (43%)	9 (24%)	
> 10 years	24 (57%)	28 (76%)	
Employment duration in the flight operation area or in the hangars			
Did not work in that area or less than 1 year	6 (14%)	25 (68%)	< 0.0001^b)^
1–10 years	16 (38%)	7 (19%)	0.357^c)^
> 10 years	20 (48%)	5 (14%)	
Share of working hours in the operative area or in the hangars			< 0.0001
Rarely or never	7 (17%)	29 (78%)	
1/4 to 1/2 of the time	13 (31%)	4 (11%)	
Almost all the time	21 (50%)	4 (11%)^d)^	
Missing	1 (2%)	0	
Share of working hours exposed through skin to soot or jet fuel			< 0.0001
Rarely or never	14 (33%)	33 (89%)	
1/4 to 1/2 of the time	16 (38%)	≤ 3^e)^	
Almost all the time	12 (29%)	≤ 3^e)^	
Missing	0	1 (3%)	
Smoking			0.197
Occasional or former smoker	18 (43%)	9 (24%)	
Never smoker	23 (55%)	27 (73%)	
Missing	1 (2%)	1 (3%)	
Smokeless tobacco (snus)			0.532
Current user	0	0	
Used in the past	≤ 3^c)^	0	
Never used	39 (93%)	36 (97%)	
Missing	1 (2%)	1 (3%)	
Exposed to passive smoking at home or work	6 (14%)	≤ 3^c)^	0.587
Missing	2 (3%)	1 (2%)	
Alcohol intake per week day (alcohol units)^f)^			0.355
0	32 (76%)	24 (65%)	
1–2	6 (14%)	10 (27%)	
3–4	0	0	
5	0	0	
Missing	4 (10%)	3 (8%)	
Sun bath in the last 3 days^g)^			0.608
Yes	4 (10%)	6 (16%)	
No	37 (88%)	31 (84%)	
Missing	1 (2%)	0	
Health history			
Relevant for lung function (asthma)	≤ 3 c)	≤ 3^e)^	
Relevant for inflammation markers (diabetes and/or eczema)	6 (14%)	≤ 3^e)^	
Relevant for DNA damage (cancer, diabetes and/or stroke)	≤ 3^c)^	≤ 3^e)^	
Other (migraine and unspecified)	4 (10%)	6 (16%)	
Having cold-like symptoms in the last 7 days			0.531
No	35 (83%)	31 (84%)	
Have had or currently having	7 (17%)	6 (16%)	
Medication intake in last 14 days^h)^			0.007
Anti-histaminic and analgesic	5 (12%)	14 (38%)	
Anti-inflammatory	0	≤ 3^e)^	
Blood pressure and cholesterol	6 (14%)	4 (11%)	
Others and missing drug specification	7 (17%)	≤ 3^e)^	

Data presented as average (standard deviation) or number (percentage).

Reference group includes military staff working as office workers (31) and avionics (6). Exposed group includes crew chiefs (17), aircraft engineers (14), fuel operators (6) and munition specialists (5). Missing values from categorical variables were eliminated in the determination of p value. The p values were determined by Chi-square test.

^a)^BMI, body mass index, calculated from the reported weight and height and considering BMI ≥ 25 < 30 as overweight and BMI $\ge$ 30 as obese.

^b)^p value for the question if they performed work at the operative area or hangar.

^c)^p value for the question about the work-years length in the operative flight area (n = 52).

^d)^Office workers with the office located in the hangar.

^e)^When the number of cases is equal or below 3, for confidentiality reasons, data are not presented.

^f)^One alcohol unit was considered to be equivalent to 1 flask of beer, 1 glass of wine or 4 cl. of spirit.

^g)^Does not include solarium use (a specific question on that matter had no positive answers and 3 missing).

^h)^Self-reported medication grouped by action classes. The p value for medication intake was determined to a dichotomised variable *yes/no* for intake from all drugs.

### Exposure and effect assessment

Measurement of personal exposure to UFP failed recording for four participants due to technical problems, with results limited to 9 participants as presented in Table 2. The two crew chief’s measurements present the highest averages and percentiles among the measured job functions. Moreover, their arithmetic mean is higher than the 90^th^ percentile, reflecting that short period peaks dragged the average for the entire data set higher. As described above, the measurements were not collected during a full working day. Therefore, we also included a time-series of a crew chief from a previous campaign at the same site capturing two full cycles of a normal workflow previously reported in^8^. No further associations of UFP with biomarkers were possible.

**Table 2:** Personal ultrafine particle exposure measured by job function (from time series data).

Job function	Place	Time (min)	Average number concentration ± SD (#/cm^3^)	Number concentration 10th and 90th percentiles (#/cm^3^)	Average size (nm)
Crew chief (n = 2/17)	Hangar	253	13.8 × 10^3^ ± 88.1 × 10^3a)^	1.6 × 10^3^ to 11.1 × 10^3b)^	58
Aircraft engineer (n = 1/14)	Workshop	297	1.9 × 10^3^ ± 1.7 × 10^3^	0.8 × 10^3^ to 2.9 × 10^3^	106
Office worker (n = 6/31)	Office	223	2.4 × 10^3^ ± 9.2 × 10^3a)^	0.4 × 10^3^ to 4.4 × 10^3b)^	60
Previously published ^8^ Crew chief (n = 1)^c)^	Hangar	364	51.6 × 10^3^ ± 330.5 × 10^3^	4.9 × 10^3^ to 30.1 × 10^3^	61

SD, standard deviation.

^a^^)^Pooled standard deviation from the different time series (square root of averaged variance).

^b^^)^Pooled percentiles from all data from the different time series.

^c^^)^Breathing zone from crew chiefs measured in the same air base in May 2017.

**Figure 1:**
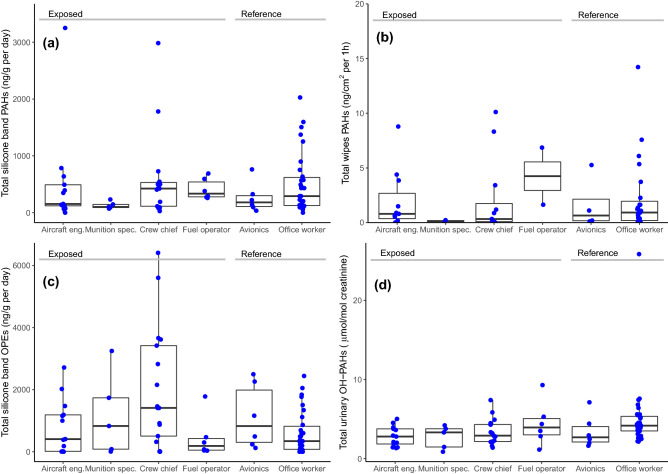
Exposure markers levels by job function: (**a**) PAHs in silicone bands (n = 77); (**b**) PAHs in skin wipes (n = 54), (**c**) OPEs in silicone bands (n = 77) and (**d**) OH-PAHs in urine (n = 78); Dots represent individual measurements and boxplot represent median and interquartile range (25–75%). PAHs, polycyclic aromatic hydrocarbons; OPEs, organophosphate esters; OH-PAHs, monohydroxylated metabolites of PAHs.

**Figure 2:**
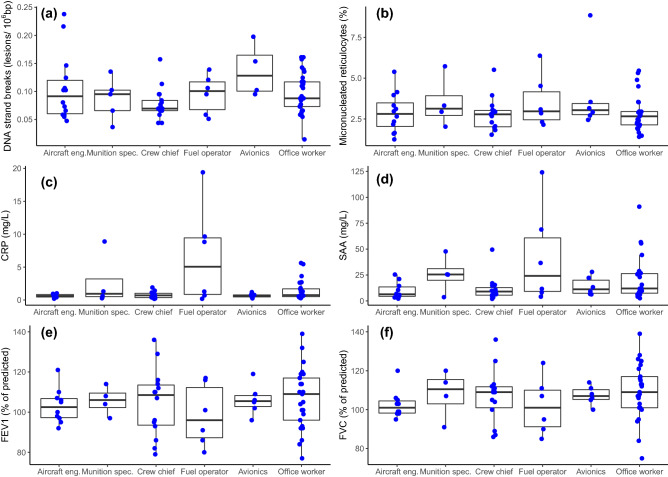
Effect marker levels per job function: (**a**) DNA strand breaks (n = 77); (**b**) Frequency of micronucleated + CD71 reticulocytes (n = 70); (**c**) Inflammation CRP (n = 65); (**d**) Inflammation SAA (n = 65); (**e**) Lung function FEV1 (n = 65); (**f**) Lung function FVC (n = 65). Dots represent individual measurements and boxplots represent interquartile range (25–75%). CRP, C-reactive protein; SAA, serum amyloid A; FEV1, forced expiratory volume in 1 s; FEV, forced vital capacity.

Figures 1 and 2 present the markers of exposure and effect distributions, respectively, per job function. The exposure markers in Fig. 1 are shown as best estimates of total amounts in the different matrixes (silicone bands, skin wipes and urine) with complementary information on individual compounds and isomers analysed (15 PAHs, 7 OPEs and 7 OH-PAHs) in the supplement (Tables S6-S12 and Figures S5-S7). In the supplement, we also report the aggregated data under three different analytical criteria for values under LOQ: best estimates, 1/2 LOQ and zeros (Tables S6, S8 and S9).

The exposure markers presented as total amounts show high variations and overall not presenting consistent differences across job functions, which may also reflect the small sample sizes. For PAHs, the correlations between PAH levels measured in different matrixes were weak (Supplementary Figure S8). Most of the individual compounds (supplementary Tables S6-S12, and Figures S5-S7) had the same high levels of variance and lack of consistent differences. For a few individual PAHs and OPEs in silicone bands, there was a statistical mean difference between exposure groups. This was found for the PAH fluorene (F = 7.6; p = 0.007) with mean (± SD) group levels of 15.9 ± 23.7 and 5.28 ± 7.87 ng/g per 24 h, for exposed and reference group, respectively, and the OPE TPHP (W = 966.5, p value = 0.011), with mean (± SD) values of 305 ± 606 and 19.7 ± 33.8 ng/g per 24 h for exposed and reference groups, respectively. The OPE TDCIPP had higher mean in the exposed group (60.7 ± 135 ng/g per 24 h) compared to the reference group (8.89 ± 15.7 ng/g per 24 h) but did not reach statistical significance (W = 825.5, p value = 0.286). TPHP and TDCIPP were correlated (r = 0.75, p < 0.0001, Figure S9). The urinary excretion of 2-FLU was not predicted by the silicone content of fluorene (F = 0.019; p = 0.890). Specifically, silicone band levels of fluorene were higher for fuel operators (Supplemental Figure S5), and the levels of two specific OPEs (TPHP and TDCIPP) were higher for crew chiefs (Supplemental Figure S6). The levels of PAHs and OPEs measured in silicone bands and urinary OH-PAHs varied also between campaign days (Tables S11 and S12). Skin wipes have low number of samples per job function and short period of exposure, and were excluded from further analysis.

Participants who reported to have or have had cold-like-symptoms in the last week were excluded from inflammatory (CRP and SAA) and lung function (FEV1, FVC and PEF) endpoint analyses (n = 13), but not from genotoxicity analysis^18^. Additionally, participants who reported to have taken anti-inflammatory drugs in the last 15 days were excluded from inflammatory endpoint analysis.

### Association between exposure and biomarkers

With the test for equal means, the defined reference and exposed groups were not significantly different in any of the exposure or effect markers, except for the urinary excretion of PAH metabolites, which was lower for the exposed group as compared to the reference. However, the difference was no longer significant when excluding females (Table 3 and Table S13). The multivariate analysis of means for the two groups by Hotelling T-squared test did not show differences for the inflammatory markers SAA and CRP (T = 1.51; p = 0.229), DNA strand breaks and micronuclei frequency (T = 1.22; p = 0.302) or lung function FVC, FEV1 and PEF (T = 0.79; p = 0.501).

**Table 3:** Average (± SD) of the exposure and effect endpoint levels between the defined reference and exposed groups and p value from analysis of variance test.

Biomarker	Exposed group	N = 42	Reference group	N = 37	*p* value^a^^)^
Silicone bands total PAHs (ng/g of band per day)	479 (± 683)	41	473 (± 503)	36	0.783
Silicone bands total OPEs (ng/g of band per day)	1311 (± 1552)	41	700 (± 766)	36	0.190
Skin wipes total PAHs (ng/cm^2^ per 1 h)^b)^	2.05 (± 3.02)	27	2.10 (± 3.20)	27	0.718
Total urinary OH-PAHs (µmol/mol creatinine)	3.29 (± 1.7)	41	4.76 (± 3.9)	37	**0.004**
DNA strand breaks (number of lesions/10^6^ bp)	0.09 (± 0.04)	42	0.10 (± 0.04)	35	0.202
Micronucleated + DC71 reticulocytes (‰)	3.01 (± 1.2)	37	3.04 (± 1.4)	33	0.990
CRP (mg/L)	1.96 (± 3.9)	35	1.29 (± 1.4)	30	0.832
SAA (mg/L)	17.84 (± 23.9)	35	20.15 (± 19.6)	30	0.211
FEV1 (% of predicted)	103 (± 13)	34	107 (± 14)	31	0.322
FVC (% of predicted)	105 (± 12)	34	109 (± 13)	31	0.176
PEF % of predicted)	119 (± 17)	34	120 (± 20)	31	0.812

bp, base pairs; CRP, C-reactive protein; FEV1, forced expiratory volume in 1 s; FVC, forced vital capacity; OH-PAHs, monohydroxylated metabolites of PAHs; OPE, organophosphate esters; PAHs, polycyclic aromatic hydrocarbons; PEF, peak expiratory flow; SAA, serum amyloid A; SD, standard deviation.

^a^^)^The p values were determined by the Welch one-way test on normal data (transformed when needed, as reported in statistics description, and eliminating zeros when log-transformed).

^b^^)^Skin wipe data corresponds to the sum of left, right and neck wipes normalized for 1 h.

Table 4 presents the output results from linear regression models for each of the effect endpoints, considering the factorial exposure group definition adjusted for identified potential confounders.

**Table 4:** Regression analysis for biomarkers of effect, using exposure group, age, sex, BMI, relevant health history, and for lung function also smoking history as predictors.

Explained variable	N = 79	R^2^	Model p value	Predictors	Parameter estimate	SE	p value
DNA strand breaks	77	0.048	0.613	Exposure group	− 0.363	0.269	0.182
				Age	− 0.003	0.013	0.817
				Sex (male)	0.346	0.397	0.386
				BMI	0.041	0.039	0.294
				Health history (diabetes, stroke, cancer)	0.173	0.539	0.749
				Intercept	1.520	1.235	0.223
Natural logarithm of micronucleated + CD71 reticulocytes	70	0.021	0.923	Exposure group	− 0.020	0.100	0.839
				Age	0.001	0.005	0.799
				Sex (male)	0.150	0.156	0.341
				BMI	0.007	0.015	0.618
				Health history (diabetes, stroke, cancer)	− 0.082	0.190	0.669
				Intercept	0.658	0.464	0.161
Natural logarithm of CRP	64	0.263	0.003	Exposure group	0.043	0.233	0.853
				Age	− 0.019	0.012	0.110
				Sex (male)	− 0.387	0.352	0.277
				BMI	0.138	0.033	** < 0.0001**
				Health history (diabetes and eczema)	− 0.148	0.386	0.703
				Intercept	− 2.704	1.083	0.015
Natural logarithm of SAA	64	0.299	0.001	Exposure group	− 0.043	0.213	0.839
				Age	− 0.004	0.011	0.706
				Sex (male)	− 1.110	0.322	**0.001**
				BMI	0.105	0.030	**0.001**
				Health history (diabetes and eczema)	− 0.487	0.352	0.172
				Intercept	0.856	0.988	0.390
FEV1^a^^)^	63	0.051	0.546	Exposure group	− 3.689	3.538	0.301
				BMI	− 0.647	0.499	0.200
				Health history (asthma)	1.955	14.01	0.890
				Smoke history (occasional or former smoker)	2.416	3.654	0.511
				Intercept	124.02	14.07	< 0.0001
FVC^a^^)^	63	0.055	0.504	Exposure group	− 4.226	3.192	0.191
				BMI	− 0.479	0.450	0.292
				Health history (asthma)	3.789	12.72	0.767
				Smoke history (occasional or former smoker)	2.352	3.297	0.478
				Intercept	121.78	12.69	< 0.0001
PEF^a^^)^	63	0.045	0.604	Exposure group	− 0.121	4.770	0.980
				BMI	− 0.200	0.673	0.767
				Health history (asthma)	28.50	19.01	0.139
				Smoke history (occasional or former smoker)	3.489	4.926	0.482
				Intercept	123.75	18.97	< 0.0001

BMI, body mass index; CRP, C-reactive protein; FEV1, forced expiratory volume in 1 s; FVC, forced vital capacity; OH-PAHs, monohydroxylated metabolites of PAHs; OPE, organophosphate esters; PAHs, polycyclic aromatic hydrocarbons; PEF, peak expiratory flow; R^2^, proportion of variance explained by the model; SAA, serum amyloid A; SE, standard error.

^a^^)^Lung function variables (FEV1, FVC and PEF) are percentage of predicted values according to sex, age and height, and therefore here not adjusted for age and sex.

The exposure (as factorial exposure group definition), was not statistically associated with any of the endpoints analysed, with the model significant p values attributable to the significant influence of BMI and sex. The analysis excluding avionics and 4 office workers with office located in the hangar did not change significantly the associations (Tables S14 and S15).

## Discussion

We performed a cross-sectional study of employees at a Danish military air base. We measured exposure levels to PAHs, OPEs, and urinary OH-PAHs. Additionally, we assessed exposure levels of UFP in breathing zone of workers for specific job functions. As biomarkers of effect, we assessed lung function, plasma levels of acute phase proteins and genotoxicity in peripheral blood cells. Overall, the levels of markers of exposure and effect found in the current study were not different between the exposed and reference groups.

The use of PPE in this study may reflect an increased use of exposure prevention measures, as compared to a historical study in Danish air force bases where no gloves, masks or protective clothing were used by fuel operators^19^. Differences of PPE use could be seen across different job functions, which may reflect variation in their different job tasks and working location rather than compliance with protective procedures, especially concerning the use of mask. Unfortunately, we were unable to assess the effect of PPE use, as we did not collect data on PPE use related to specific tasks.

Particle exposure was assessed using personal monitors. In order to increase the exposure assessment of crew chiefs, we included data from our recent exposure study at the same air base^8^. The UFP breathing zone measurements of a crew chief, monitored during two cycles of aircraft leaving, arriving and being fuelled by a truck, within 6 h, had higher exposure levels^8^ than in the present study. Monitoring time and daily variation are likely to explain this difference. In the present study, we monitored two crew chiefs for 4 h and 4h30 without controlling for the aircraft movement cycles. The measurements from both studies were performed in the spring (of 2017 and 2018), but daily variations in weather conditions and operation activities are other factors of variability. In a study from an Italian aviation base, UFPs were measured in the breathing zone of a crew chief for 10 consecutive full working days with an equivalent device (Nanotracer) showing higher exposure levels than reported here^20^. Furthermore, the personal exposure levels were higher compared to exposure levels measured by stationary monitors^20^. Buonanno et al. measured medians 9 times higher with 75th percentile 17 times higher than what we measured for crew chiefs in this study (and 4 × and 12 × than previously reported^8^). The aviation base in the Buonanno et al. study had a large number of aircraft and ground vehicle activities (with 6000 workers and 30,000 activities during a year), besides the location site difference and higher background levels^20^. Nevertheless, the UFP levels recorded in our studies have a marked erratic behaviour, characterized by short-time (10–20 min) high peak levels reaching maximum values of 10^6^ particles/cm^3^ similar to Buonanno et al. maximum UFP levels^20^. This may reflect that the crew chiefs in our study were close to less aircraft movements.

Total PAHs, assessed through silicone bands and skin wipes, were not different between our defined exposure groups, except for fluorene in silicone bands, where the difference was driven by fuel operator’s exposure. Nevertheless, silicone band fluorene was not associated with the corresponding urinary metabolite 2-FLU. Fluorene was not detected in any skin wipe samples (neither in hands nor in neck samples). Similarly, naphthalene in skin wipes had very few samples above LOQ (9% in neck and 2% in hand wipes), therefore it was not possible to do any correlation analyses. Napthalene levels were not different between our exposure groups, neither was the excretion metabolite 1-NAP nor 2-NAP. Levels of naphthalene measured in silicone bands were not associated with excretion of napthols (total or individually). Despite using imputed values for < LOQ based on the best estimates, the statistical analyses of the PAHs is likely associated with larger uncertainties due to the relatively large fraction of imputed data. Our OH-PAH levels are much lower than reported in previous studies on occupational exposure to jet fuel, which reported higher exposure to naphthalene associated with urinary excretion of naphtols^21–25^. In a repeated measurement study on US air force personnel, Serdar et al., observed elevated and correlated post exposure levels among naphthalene in air, breath and urinary naphtols, after a shift work of 4 h^24^. The same research group further investigated the contributions of dermal and inhalation exposures to naphthalene and urinary naphtols, observing that the dermal exposure was associated with levels of 2-NAP but not 1-NAP^21^. Rodrigues et al., performed another jet fuel exposure study among US air force personnel, with repeated pre and post shift samples from high exposed vs low exposed subjects. In that study, elevated creatinine adjusted (which allows comparison with the current study) levels of 1-NAP, 2-NAP and 2-FLU, and decreased levels of 1-hydroxypyrene (1-PYR) were observed in the higher exposure group^23^. The levels of 1-NAP, 2-NAP and 2-FLU in our exposed group were: 727 ng/g creatinine for 1-NAP; 2739 ng/g creatinine for 2-NAP; and 184 ng/g creatinine for 2-FLU, respectively. These levels were lower than the levels reported for the higher exposed workers from Rodrigues et al., and within the range of the exposure levels reported for their low exposed workers^23^. This might suggest that our study participants were exposed to jet fuel at a lower degree. However, it cannot be ruled out that the timing of sampling may also be important when comparing between studies as the window of excretion of OH-PAHs following exposure is quite narrow^26^. Sex differences might be expected for urinary markers due to lower female excretion of creatinine, and this may affect the reported adjusted levels. In fact, when removing female participants, the average levels of the creatinine-adjusted metabolites in the exposed group increased slightly, but were still lower than reported by Rodrigues et al., 2014, who had 97% male subjects in the higher exposed group^23^. The urinary metabolite levels of individual OH-PAHs were not different between job functions, but 1-PHE, 2,3-PHE and 1-PYR were significantly different between campaign days, which together with the non-significantly higher levels in our reference group, may suggest other PAHs concurrent exposures. Overall, low PAH exposure levels were found by all measures (i.e. silicone bands, skin wipes and in urinary excretion). The low exposure levels may also contribute for the lack of correlation between silicone bands and urine levels.

Total OPE levels were higher in the exposed group compared to the reference group, although the difference did not reach statistical significance. However, TPHP individually, was significantly higher in silicone bands worn by the workers in the exposed group. This was driven by crew chiefs’ exposure levels, although TPHP was not the most prevalent OPE found in these samples (which was TMPP mix of isomers). TPHP has been widely found in general population exposure^27^. Hammel et al. measured levels of OPEs in silicone bands worn by 40 subjects recruited from a university campus for 5 full days, including sleeping and bathing, reporting a median of 395 ng/band TPHP with a maximum level of 1841 ng/band^28^. In our study, the TPHP median levels for crew chiefs were 434 ng/g normalized for 24 h with maximum level of 2584 ng/g per 24 h (corresponding to approximately median levels of 2300 ng/band and maximum level of 13,700 ng/band with equivalent band weights in both studies), which is considerably higher taking the shorter sampling period into account (1 day vs 5 days), in turn suggesting occupational exposure. A recent study investigated OPEs levels among US military aircraft maintainers, also using silicone bands as passive samplers, measuring an average of 1.67 ng/g of TNBP, 832.4 ng/g of TPHP, and 2352.1 ng/g of TMPP for a shift work of crew chiefs (n = 14)^29,30^, showing a similar trend in prevalence and level as the crew chiefs in our study (mean exposure levels were 49 ng/g TNBP, 622 ng/g TPHP and 959 ng/g TMPP, respectively). However, in the Hardos et al. report, crew chief was not the job function with the highest mean levels for any of these three OPEs assessed, since aerospace propulsion, fuel system repair and avionics presented higher or equivalent mean levels for each of the OPEs^29^. Overall, Hardos et al. suggested that the use of job functional career code system for aircraft maintenance employees was not a good predictor of exposure^30^.

Sex differences for effect biomarkers of acute-phase reactants, genotoxicity and lung function could be expected^31–33^, but no significant differences were observed between the defined groups though the sex composition was marginally different. BMI and sex are known predictors of SAA and CRP levels^34^. From the regression analysis, none of the biological endpoints assessed was predicted by the exposure group defined, and the major relative predictor of the different endpoints (reaching significance for inflammation markers) were sex (being a male) and BMI. Thus, we were able to reproduce known predictors of SAA and CRP (BMI, Figure S10). Our study participants, being military staff, need to pass a test for physical fitness, which may influence their levels of CRP, SAA and lung function^35^.

The comet assay is virtually a standard method for the assessment of DNA damage by environmental and occupational exposures^32^. We have previously demonstrated that controlled exposure to particulate matter from diesel engines or firefighter activities was associated with elevated levels of DNA damage in PBMCs^9,10^. However, a field study among firefighters did not indicate genotoxicity in PBMCs after a work shift, which was considered to be related to both low exposure during the work shift under study and possibly carry-over from previous exposures, even though the firefighters were monitored after three days off^36^. Previous studies on jet fuel exposure among aircraft and airport workers assessed genetic damage by sister chromatic exchange (SCE), micronuclei frequency, chromosomal aberrations, DNA strand breaks and oxidatively damaged DNA with inconsistent results^22,37–40^. Lemasters et al.^37^ assessed genotoxic changes in aircraft maintenance personnel exposed to solvents and jet fuel (mostly JP-4) in a sequential study, and reported a small but statistically significant increase in the frequency of SCE of a group of workers after 30 weeks of exposure, while observing a non-statistically significant increase in micronuclei frequency in the unexposed group. SCE frequency was also observed to be higher in the exposed group of military air force workers in a cross-sectional study from Erden et al. accompanied by a no difference in micronuclei frequency^22^. Pitarque et al. did not detect changes in SCE in a cross-sectional study among airport workers, but observed lower micronuclei frequency in the exposed group, and small increase in DNA damage detected by the comet assay^38^. Cavallo et al.^40^ assessed genetic damage in lymphocytes and buccal cells of airport workers exposed to PAHs from jet fuel exhausts by SCE, micronuclei frequency, chromosomal aberrations and oxidative damage to DNA in a cross-sectional study. The assessed biomarker of exposure (urinary 1-PYR) was not different among exposure groups, but they observed higher frequencies of SCE, chromosomal aberrations and oxidative damage to DNA in the exposed group, while no difference was found for the micronuclei frequencies in both lymphocytes and buccal cells^40^. Krieg et al.^39^ investigated DNA strand break levels in leukocytes from pre- and post-4 h-work-shift samples from air force personnel exposed to jet fuel, using exposure group, or benzene and naphthalene breathing zone and exhaled breath measurements as well as their urinary markers, as predictors. They did not observe genotoxic effects assessed by the comet assay^39^. The levels of DNA strand breaks in both exposed and reference group (approximately 0.1 lesions/10^6^ bp) were similar to what we previously measured in firefighters before and after a shift work (0.13 lesions/10^6^ bp)^36^, in young conscripts before and after firefighting exercises (0.2 lesions/10^6^ bp)^9^ and in volunteers travelling in electric and diesel trains (0.12 and 0.18 lesions/10^6^ bp)^10^, analysed in different laboratories and using different calibration curves.

We observed a potential occupational exposure to two OPEs (TPHP and TDCIPP), which was significantly different between the defined exposure groups for TPHP, and driven by the measured crew chief levels (as shown in supplementary Figure S6). TDCIPP levels were also higher in the exposed group, driven by crew chiefs, but did not reach a statistical difference. However, the extremely skewed data with an excess of zeros (68% of zeros), may represent an exposure level that did not occur for a large percentage of the participants, but relatively large values (95th percentile of 249 ng/g per 24 h with max 588 ng/g per 24 h) were measured in the group of crew chiefs, suggesting that the small sample size limited our observation. The median of TPHP, TDCIPP and 75th percentile of TDCIPP for crew chiefs were markedly higher than observed by Hammel et al.^28^ for members of the general U.S.-population (assuming approximate equal wristband sizes). TDCIPP is a chlorine-containing mix of OPE isomers, with observed carcinogenicity in rats by oral route, mutagenicity data showing no genotoxic effects and overall with limited evidence of carcinogenic effect for humans^41,42^. TPHP is not considered to be mutagenic^43^. Interestingly, in a cytotoxicity screening study of binary mixture interactions between emerging environmental organic compounds, the pair TPHP and TDCIPP exhibited one of the most significant synergistic effects that prompted a transcriptome and metabolome characterization, showing significant effects, namely for oxidative stress and affected purine and pyrimidine metabolism^44^. Nevertheless, even though the exposure to this pair of OPEs was observed as significantly elevated for the exposed group, driven by crew chiefs, there was no difference for the two biomarkers of genotoxicity.

Jet fuel subacute exposure demonstrated limited effect on the airways or immune system in an animal model^45^. TPHP and TDCIPP might have potential for immunotoxicity, observed on murine dendritic cells in vitro^46^. However, none of the assessed biomarkers of inflammation, CRP and SAA, showed differences between the exposure groups. Nevertheless, the cross-sectional design, the small sample size, the complexity of the exposure and the fact that the reference group may also have some background exposure, might have limited our observations.

## Conclusions

Overall, our results show limited evidence of occupational exposure of the studied air force ground personnel measured through UFP, PAHs, OPEs and urinary OH-PAHs. Total PAHs and OPEs assessed in silicone bands were observed not to be significantly different between our exposed and reference groups. However, individual chemicals were significantly different between the groups with the PAH fluorene being higher for fuel operators and the OPE TPHP observed to be significantly higher for the exposed group, driven by crew chiefs. The OPE TDCIPP was also measured to be higher for the crew chiefs, although not statistically significant. When comparing the exposed group with the reference group, there was no evidence of difference in biological effects assessed through markers of systemic inflammation (SAA and CRP), genetic damage (DNA strand breaks or micronuclei) or lung function (FEV1, FVC or PEF).

## Supplementary Information

Supplementary Information.

## Data Availability

The datasets analysed during the current study are available as aggregated data in manuscript and supplementary tables and further available from the corresponding authors on reasonable request.

## Abbreviations

1-NAP: 1-Hydroxynapthalene
2-NAP: 2-Hydroxynapthalene
2-FLU: 2-Hydroxyfluorene
1,2,3,4-PHE: 1,2,3,4-Hydroxyphenanthrene
1-PYR: 1-Hydroxypyrene
CRP: C-reactive protein
DAG: Directed acyclic graph
FEV1: Forced expiratory volume in one second
FVC: Forced vital capacity
JP-8: Jet propulsion fuel 8
OH-PAHs: Monohydroxylated metabolites of PAHs
OPE: Organophosphate ester
PAH: Polycyclic aromatic hydrocarbon
PBMC: Peripheral blood mononuclear cells
PBS: Phosphate-buffered saline
PEF: Peak expiratory flow
PPE: Personal protective equipment
SAA: Serum amyloid A
TPHP: Triphenyl phosphate
TMPPMIX: Tri(methyl phenyl) phosphate mixture of isomers
TMPP: Tris(2-methylphenyl) phosphate
TNBP: Tri-*n*-butyl phosphate
TCEP: Tris(2-chloroethyl)phosphate
TCIPP: Tris(chloroisopropyl)phosphate
TDCIPP: Tris(1,3-dichloro-2-propyl)phosphate
UFP: Ultrafine particles
