# Supplementary material for: Occupational exposure and markers of genetic damage, systemic inflammation and lung function: a Danish cross-sectional study among air force personnel
Source: Sci Rep. 2021 Sep 9;11:17998. doi: 10.1038/s41598-021-97382-5 (PMC8429754; doi:10.1038/s41598-021-97382-5)
Supplement: Supplementary file 1 — Supplementary Information. [file 41598_2021_97382_MOESM1_ESM.pdf]

SUPPORTING INFORMATION TO THE MANUSCRIPT

Occupational exposure and markers of genetic damage, systemic inflammation and lung function: a Danish cross-sectional study among air force personnel

Maria Helena Guerra Andersen[1], Anne Thoustrup Saber[1], Marie Frederiksen[1], Per Axel Clausen[1], Camilla Sandal Sejbaek[1], Caroline Hallas Hemmingsen[2], Niels Ebbehøj[2], Julia Catalán[3,4], Kukka Aimonen[3], Joonas Koivisto[1,5], Steffen Loft[6], Peter Møller[6] and Ulla Vogel[1,7*]

[1]The National Research Centre for the Working Environment, Lersø Parkalle 105, DK-2100 Copenhagen Ø, Denmark. [2]Department of Occupational and Environmental Medicine, Bispebjerg Hospital, Bispebjerg Bakke 23, DK-2400 Copenhagen NV, Denmark. [3]Finnish Institute of Occupational Health, P.O. Box 40, FI-00032 Työterveyslaitos, Finland, [4]Department of Anatomy, Embryology and Genetics, University of Zaragoza, 50013 Zaragoza, Spain. [5]ARCHE Consulting, Liefkensstraat 35D B-9032 Wondelgem, Belgium. [6]Department of Public Health, Section of Environmental Health, University of Copenhagen, Øster Farimagsgade 5A, DK-1014 Copenhagen K, Denmark. [7]Department of Health Technology, Technical University of Denmark, DK-2800 Kgs. Lyngby, Denmark.

Content

Tables	Title	Page
**Table S1**	Individual chemicals analyzed, limit of quantification (LOQ) and percentage of samples quantified for each matrix	3
**Table S2**	Results from negative and positive comet assay controls. Data is presented as average and standard deviation	4
**Table S3**	Results from micronuclei assay. Data presented as average and standard deviation	7
**Table S4**	Detection frequency, data missing/loss and data transformation to achieve normality (including females) used in ANOVA and Hotelling T-squared test	8
**Table S5**	Self-reported characteristics and use of personal protective equipment per job function. Data presented as average and standard deviation or number and percentage	9
**Table S6**	Median [IQR] levels of individual and total PAHs and OPEs on silicone bands per exposure group with three methods of reporting values <LOQ (best estimate, ½ LOQ and zero). Values are normalized for one day (24h) of use of the silicone band	10
**Table S7**	Median [IQR] levels of individual PAHs and OPEs concentrations per job function in silicone bands. Values are best estimates normalized for one day (24h) of use of the silicone band.	11
**Table S8**	Median [IQR] levels of individual and total PAHs on skin wipes per exposure group with three methods of reporting values <LOQ (best estimate, ½ LOQ and zero). Values are normalized for 1h (to correct for the different sampling timings)	15
**Table S9**	Median [IQR] levels of individual urinary metabolites of PAHs per exposure group with three methods of reporting values <LOQ (best estimate, ½ LOQ and zero)	16
**Table S10**	Median [IQR] levels of individual urinary metabolites of PAHs per job function and excluding females. Values are best estimates	16
**Table S11**	Median [IQR] levels of individual PAHs and OPEs in silicone bands per campaign day and per exposure group. Values are best estimates adjusted for 24h deployed time	18
**Table S12**	Median [IQR] levels of individual urinary PAHs metabolite concentration per campaign day. Values are best estimates	19
**Table S13**	Average (±SD) of the biomarker levels between the defined reference and exposure groups among male volunteers and p-value from analysis of variance test (females excluded from analysis)	21

**Table S14**	Average (±SD) of the biomarker levels between the defined reference and exposure groups without Avionics and 4 Office workers with office in the hangar. The p-value was determined from analysis of variance test	21
**Table S15**	Regression analysis for biomarkers of effect, using exposure group, age, sex, BMI and relevant health history as predictors (excluding Avionics and 4 Office workers)	22

Figures	Caption	Page
**Figure S1**	Dose response curve for the comet assay on THP1 cells exposed to X-ray (Gy of gamma rays)	4
**Figure S2**	Directed acyclic graph (DAG) for lung function (FEV1, FVC and PEF). The regression model was adjusted for age, BMI, sex, smoking history and lung related relevant health history (i.e. asthma)	5
**Figure S3**	Directed acyclic graph (DAG) for systemic inflammation (CRP and SAA). The regression model was adjusted for age, BMI, sex and inflammation related relevant health history (i.e. diabetes and eczema)	6
**Figure S4**	Directed acyclic graph (DAG) for genetic damage (strand breaks and micronuclei). The regression model was adjusted for age, BMI, sex and DNA damage related relevant history (i.e. diabetes, stroke and cancer)	7
**Figure S5**	Levels of individual and total polycyclic aromatic hydrocarbons (PAH) in silicone bands (normalized for 24h) per job function (n=77), best estimates	12-13
**Figure S6**	Levels of individual and total organophosphate esters (OPE) in silicone bands (normalized for 24h) per job function (n=77), best estimate	14
**Figure S7**	Levels of urinary creatinine and individual urinary metabolites of polycyclic aromatic hydrocarbons per job function (n=78), best estimate	17
**Figure S8**	Correlations between PAHs exposure markers	20
**Figure S9**	Correlation between two OPEs (TDCIPP and TPHP) in silicone bands	20
**Figure S10**	Inflammation markers per classes of BMI and Age. BMI, body mass index (normal: 18.5-24.9; overweight: 25.0-29.9; obese: ≥30). CRP, C-reactive protein; SAA, serum amyloid	23

Abbreviations

1-NAP, 1-hydroxynapthalene; 2-NAP, 2-hydroxynapthalene; 2-FLU, 2-hydroxyfluorene; 1,2,3,4-PHE, 1,2,3,4-hydroxyphenanthrene; 1-PYR, 1-hydroxypyrene; CRP, C-reactive protein; DAG, directed acyclic graph; FEV1, Forced respiratory volume in one second; FVC, Forced vital capacity; JP-8, Jet propulsion fuel 8; OH-PAHs, OPE, Organophosphate ester; PAH, Polycyclic aromatic hydrocarbon; PBMC, Peripheral blood mononuclear cells; PBS, phosphate-buffered saline; PEF, Peak expiratory flow; PPE, Personal protective equipment; SAA, Serum amyloid A; TPHP, Triphenyl phosphate; TMPPMIX, Tri(methyl phenyl) phosphate mixture of isomers; TMPP, Tris(2-methylphenyl) phosphate; TNBP, Tri-n-butyl phosphate; TCEP, Tris(2-chloroethyl)phosphate; TCIPP, Tris(chloroisopropyl)phosphate; TDCIPP, Tris(1,3-dichloro-2-propyl)phosphate; UFP, Ultrafine particles.

Table S1 – Individual chemicals analyzed, limit of quantification (LOQ) and percentage of samples above LOQ for each matrix

matrix	Chemical	Abbreviation or formula	Method LOQ	Detection frequency [a] (>LOQ)
Silicone bands and skin wipes	Naphthalene (CAS 91-20-3)	$C_{10}H_8$	1.6 ng/sample	Bands: 92%; wipes: 9%
	Acenaphthylene (CAS 208-96-8)	$C_{12}H_8$	5.7 ng/sample	Bands: 55%; wipes: 41%
	Acenaphthene (CAS 83-32-9)	$C_{12}H_{10}$	3.3 ng/sample	Bands 55%; wipes: 7%
	Fluorene (CAS 86-73-7)	$C_{13}H_{10}$	3.6 ng/sample	Bands: 58%; wipes: 0%
	Phenanthrene (CAS 85-01-8)	$C_{14}H_{10}$	2.7 ng/sample	Bands: 91%; wipes: 19%
	Anthracene (CAS 120-12-7)	$C_{14}H_{10}$	3.9 ng/sample	Bands: 55%; wipes: 13%
	Fluoranthene (CAS 206-44-0)	$C_{16}H_{10}$	1.9 ng/sample	Bands: 43%; wipes: 22%
	Pyrene (CAS 129-00-0)	$C_{16}H_{10}$	2.1 ng/sample	Bands: 70%; wipes: 9%
	Benz[a]anthracene (CAS 56-55-3)	$C_{18}H_{12}$	2.0 ng/sample	Bands: 49%; wipes: 44%
	Chrysene (CAS 218-01-9)	$C_{18}H_{12}$	1.4 ng/sample	Bands: 25%; wipes: 54%
	Benzo[b+k]fluoranthene (CAS 205-99-2 and 207-08-9)	$C_{20}H_{12}$	3.5 ng/sample	Bands: 49%; wipes: 15%
	Benzo[a]pyrene (CAS 50-32-8)	$C_{20}H_{12}$	4.8 ng/sample	Bands: 47%; wipes: 52%
	Dibenz[a,h]anthracene (CAS 53-07-3)	$C_{22}H_{14}$	2.5 ng/sample	Bands: 23%; wipes: 41%
	Benzo[ghi]perylene (CAS 191-24-2)	$C_{22}H_{12}$	10.1 ng/sample	Bands: 48%; wipes: 7%
	Indeno[1,2,3-cd]pyrene (CAS 193-39-5)	$C_{22}H_{12}$	2.5 ng/sample	Bands: 52%; wipes: 52%
Silicone bands	Triphenyl phosphate (CAS 115-86-6)	TPHP	3.2 ng/g	43%
	Tri(methyl phenyl)phosphate (mix of isomers) (CAS 1330-78-5)	TMPP	3.3 ng/g	65%
	Tris(2-methylphenyl) phosphate (CAS 78-30-8)	o-TMPP	3.3 ng/g [b]	35%
	Tri-n-butyl phosphate (CAS 126-73-8)	TNBP	5.3 ng/g	30%
	Tris(2-chloroethyl)phosphate (CAS 115-96-8)	TCEP	5.7 ng/g	36%
	Tris(chloroisopropyl)phosphate (mix of isomers) (CAS 13674-84-5)	TCIPP	5.9 ng/g	73%
	Tris(1,3-dichloro-2-propyl)phosphate (CAS 13674-87-8)	TDCIPP	2.6 ng/g	32%
Urinary metabolites	1-Hydroxynapthalene	1-NAP	0.27 ng/mL urine	63%
	2-Hydroxynapthalene	2-NAP	0.27 ng/mL urine	100%
	2-Hydroxyfluorene	2-FLU	0.067 ng/mL urine	95%
	1-Hydroxyphenanthrene	1-PHE	0.067 ng/mL urine	68%
	2+3-Hydroxyphenanthrene (mix of isomers)	2,3-PHE	0.067 ng/mL urine	81%
	4-Hydroxyphenanthrene	4-PHE	0.067 ng/mL urine	8%
	1-Hydroxypyrene	1-PYR	0.067 ng/mL urine	71%
Plasma markers	C-reactive protein	CRP	0.02 mg/L	100%
	Serum Amyloid A	SAA	4 ng/mL	100%

CAS, Chemical abstracts service

[a] Detection frequency for skin wipes is presented for total wipes (right hand, left hand and neck)

[b] Estimated from TMPP

Table S2 – Results from negative and positive comet assay controls. Data presented as average and standard deviation

Assay controls	Number of objects analyzed	Tail length	% DNA in tail
**Negative controls (n=5)** (A549 treated with PBS, 30 min at 4℃)	666 ± 102	19.64 ± 4.9	6.62 ± 3.1
**Positive controls (n=5)** (A549 cells treated with H_2O_2, 45 µM, 30 min at 4℃)	483 ± 52	60.27 ± 5.6	35.74 ± 2.0

PBS, phosphate buffered saline

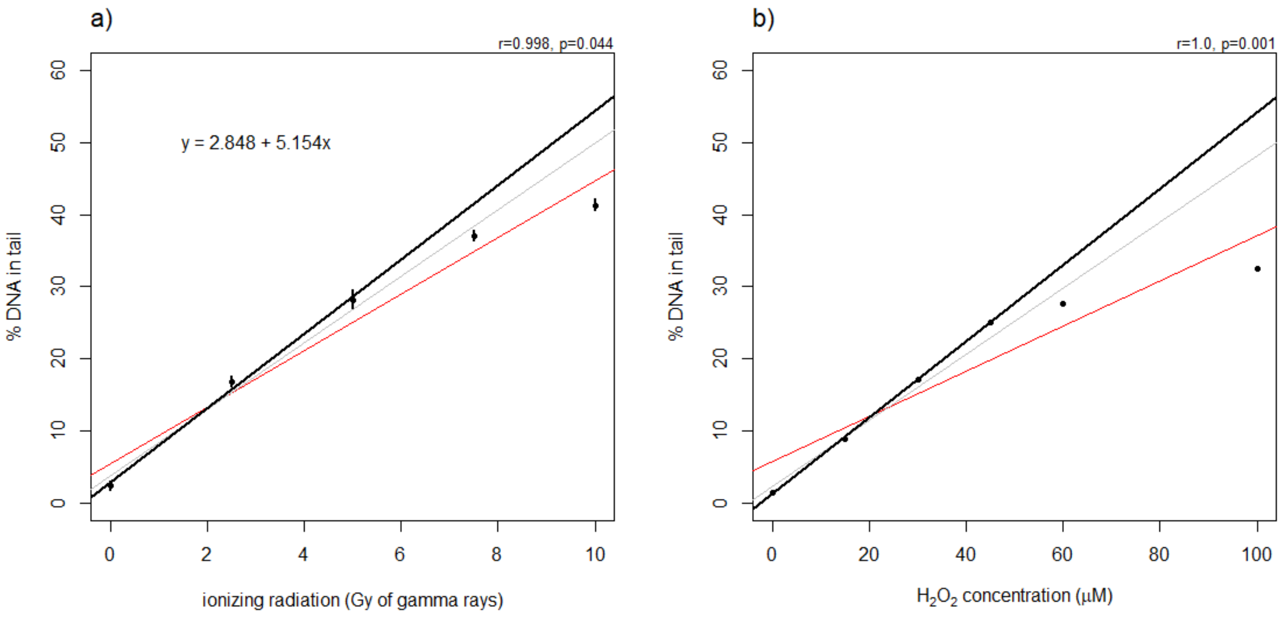

Figure S1– Calibration curve for the comet assay on THP-1 cells.

a) THP-1 cells exposed to gamma radiation (cells exposed accordingly to Forchhammer et al., 2010 [1]). Points are average of 3 technical replicates and small bars are the standard error. The black line represents the linear regression with omission of the highest two Gy's that deviate from linearity. The grey line represents the linear regression without the highest Gy and the red line with all points. The equation, the r and the p-value correspond to the black line regression;

b) THP-1 cells exposed to H_2O_2, as our assay control exposure. No replicates. The black line represent the linear regression with omission of the highest two exposure concentrations that deviate from linearity. The grey line represents the linear regression without the highest concentration and the red line with all points. The r and the p-value correspond to the black line regression;

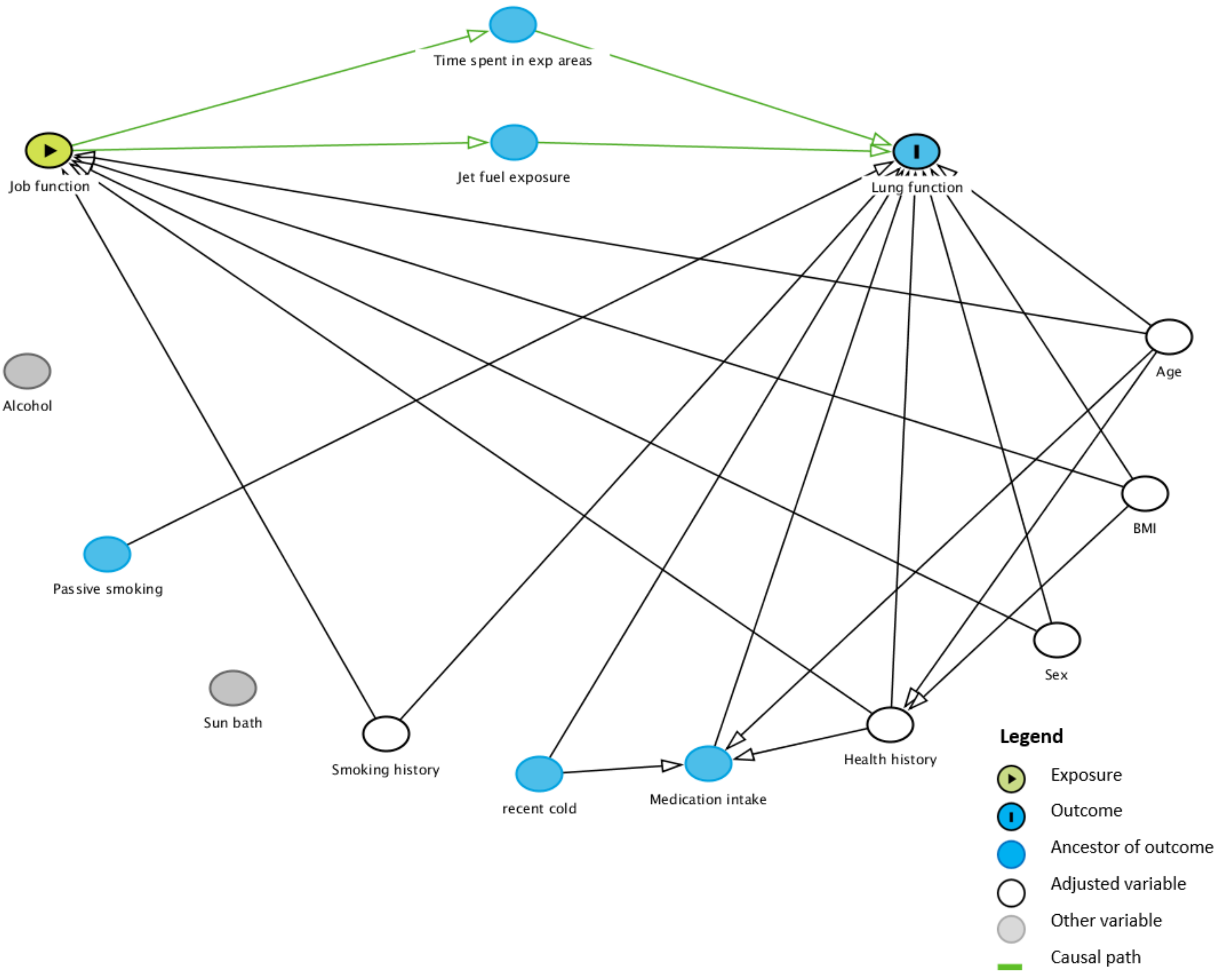

Figure S2 – Directed acyclic graph for lung function (FEV1, FVC and PEF). The regression model was adjusted for age, BMI, sex, smoking history and lung related relevant health history (i.e. asthma). BMI, body mass index; FEV1, forced expiratory volume in 1 second; FVC, forced vital capacity; PEF, peak expiratory flow.

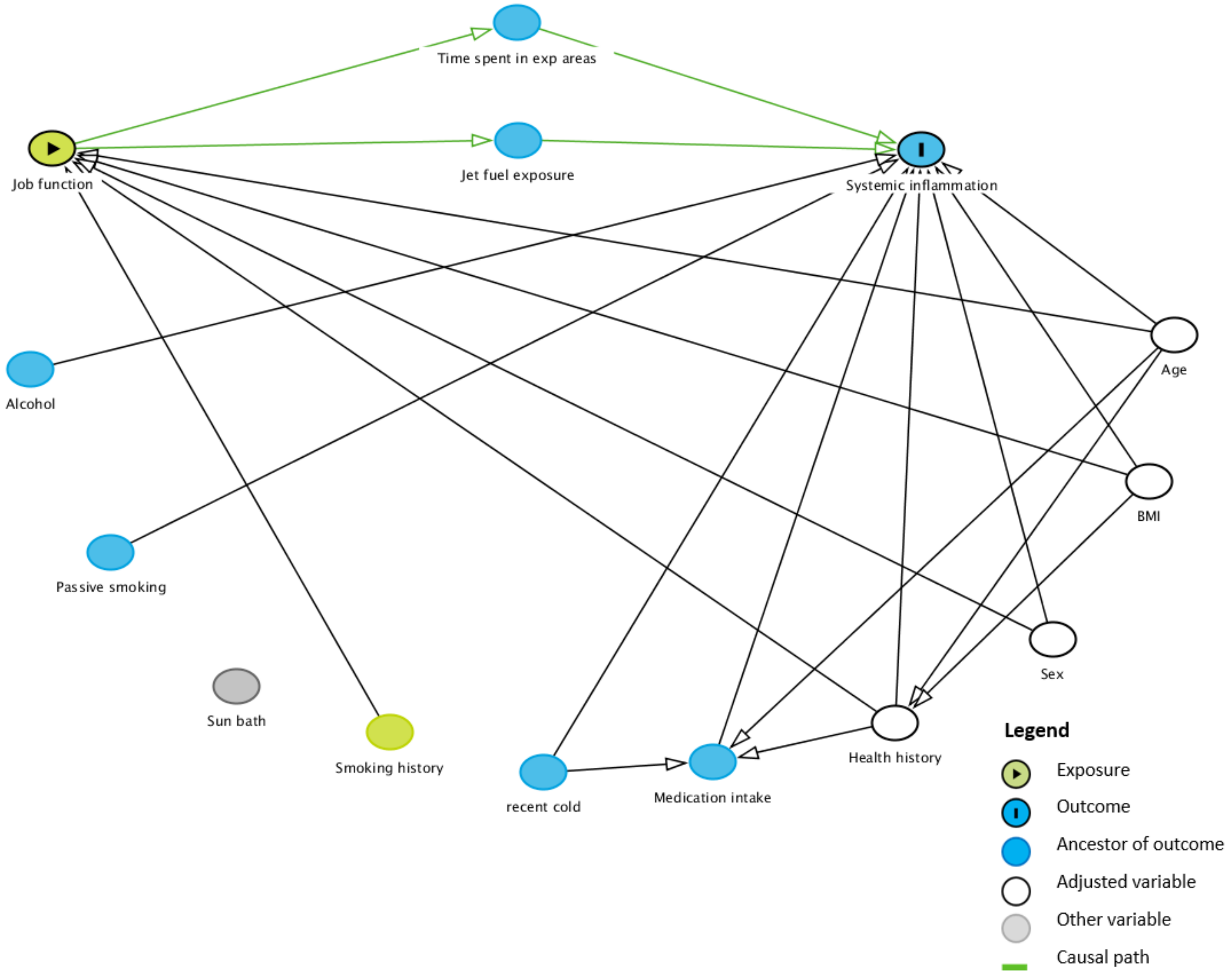

Figure S3 – Directed acyclic graph for systemic inflammation (CRP and SAA). The regression model was adjusted for age, BMI, sex and inflammation related relevant health history (i.e. diabetes and eczema). BMI, body mass index; CRP, C-reactive protein; SAA, serum amyloid A.

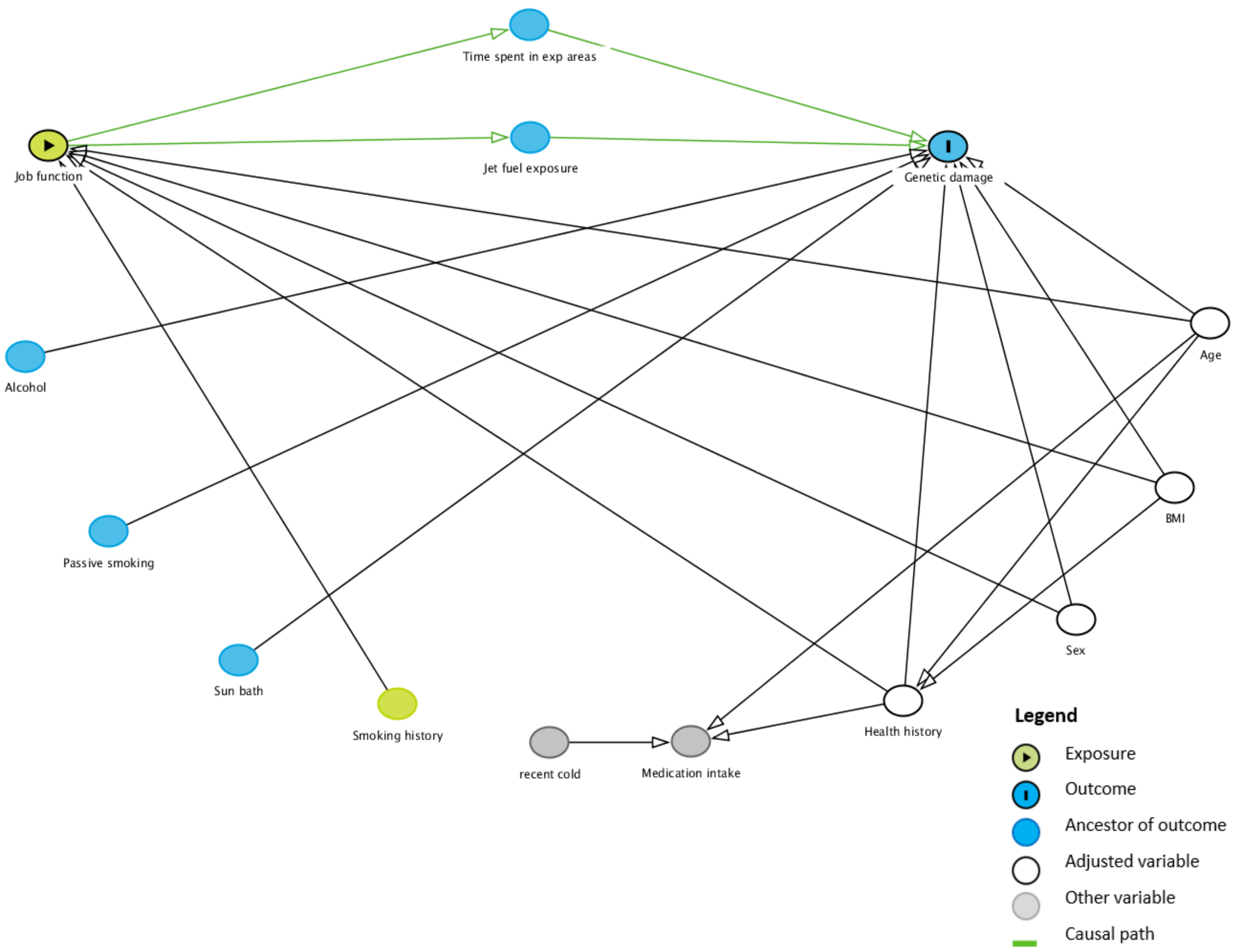

Figure S4 – Directed acyclic graph for genetic damage (DNA strand breaks and micronuclei). The regression model was adjusted for age, body mass index (BMI), sex and DNA damage related relevant history (i.e. diabetes, stroke and cancer)

Table S3 – Results from micronuclei assay. Data presented as average and standard deviation

	**Exposed group (n=37)**	**Reference group (n=33)**
**Number of analyzed +CD71 reticulocytes**	83 000 ± 23 773	74 585 ± 25 863
**Number of non-micronucleated reticulocytes**	82 770 ± 23 730	74 381 ± 25 830
**Number of micronucleated reticulocytes**	240.97 ± 109.54	204.03 ± 67.31

Table S4 – Detection frequency, data missing/loss and data transformation to achieve normality (including females) used in ANOVA and Hotelling T-squared test.

Reported results	Detection frequency (>LOQ)	Missing data and sample considerations	**Data Normality check** (Shapiro test, normal if p≥0.05) [a)]
**Total OPEs in silicone bands normalized for time of deployment**	79%	2 missing silicone bands	Raw data: not normal ($p=1.5\times10^{-9}$)<br>Logarithmic transformed data: not normal ($p=9.9\times10^{-5}$)<br>**Cubic root transformed data: normal (p=0.25)**
**Total PAHs in silicone bands normalized for time of deployment**	97%	2 missing silicone bands and 2 subjects with total PAHs=0 eliminated from analysis with log transformation	Raw data: not normal ($p=7.3\times10^{-12}$)<br>**Logarithmic transformed data: normal (p=0.45)**<br>Cubic root transformed data: not normal (p=0.012)
**Total PAH in 3 skin wipes** (left+right+neck) **normalized for time of sampling**	65%	24 missing person-skin wipes and 1 subject with total PAH=0 eliminated from analysis with log transformation	Raw data: not normal ($p= 2.4\times10^{-9}$)<br>**Logarithmic transformed data: normal (p=0.211)**<br>Cubic root transformed data: not normal (p=0.003)
**Total OH-PAHs in urine**	100%	1 missing urine sample	Raw data: not normal ($p= 1.7\times10^{-13}$)<br>**Logarithmic transformed data: normal (p=0.06)**<br>Cubic root transformed data: not normal ($p=2.7\times10^{-5}$)
**CRP in blood**	100%	2 subjects eliminated from analysis (on anti-inflammatory drugs) and subjects with cold-like symptoms in last week (n=13). Combined n=14 lost subjects	Raw data: not normal ($p=5.5\times10^{-14}$)<br>**Logarithmic transformed data: not normal ($p=5.7\times10^{-4}$)**<br>Cubic root transformed data: not normal ($p=1.6\times10^{-8}$)
**SAA in blood**	100%	2 subjects eliminated from analysis (on anti-inflammatory drugs) and subjects with cold-like symptoms in last week (n=13). Combined n=14 lost subjects	Raw data: not normal ($p=2.2\times10^{-16}$)<br>**Logarithmic transformed data: normal (p=0.07)**<br>Cubic root transformed data: not normal ($p=9.94\times10^{-8}$)
**DNA strand breaks in PBMC**	NA	2 lost samples	Raw data: not normal ($p=4.7\times10^{-4}$)<br>Logarithmic transformed data: not normal (p=0.02)<br>**Cubic root transformed data: normal (p=0.341)**
**Micronuclei frequency**	NA	9 samples excluded due to insufficient amount of +CD71 reticulocytes	Raw data: not normal ($p=4.7\times10^{-7}$)<br>**Logarithmic transformed data: normal (p=0.176)**<br>Cubic root transformed data: not normal (p=0.005)
**FVC**	NA	1 missing test; 13 subjects eliminated from analysis (with cold-like symptoms in last week)	**Raw data: normal (p=0.909)**
**FEV1**	NA	1 missing test; 13 subjects eliminated from analysis (with cold-like symptoms in last week)	**Raw data: normal (p=0.784)**
**PEF**	NA	1 missing test; 13 subjects eliminated from analysis (with cold-like symptoms in last week)	**Raw data: normal (p=0.741)**

CRP, C-reactive protein; FEV1, forced respiratory volume in 1 second; FVC, forced vital capacity; LOQ, limit of quantification; NA, not applicable; OH-PAHs, monohydroxylated metabolites of PAHs; OPEs, organophosphate esters; PAHs, polycyclic aromatic hydrocarbons; PBMC, peripheral blood mononuclear cells; PEF, peak expiratory flow; SAA, serum amyloid A. [a)] The data transformation choice used in ANOVA and Hotelling T-squared test is marked in bold.

Table S5 – Self-reported characteristics and use of personal protective equipment per exposure groups. Data presented as number and percentage.

Characteristics and use of Personal protective equipment	Exposed group (n=42)	Reference group (n=37)
Use of mask in the hangar		
Always	0	0
Often	4 (10%)	0
Sometimes	24 (57%)	0
Never	14 (33%)	35 (95%)
Missing	0	2 (5%)
Use of gloves when performing tasks with potential skin exposure		
Always	21 (50%)	8 (22%)
Often	14 (33%)	5 (14%)
Sometimes or never	7 (17%)	22 (59%)
Missing	0	2 (5%)

Table S6 – Median (IQR) levels of individual and total PAHs and OPEs on silicone bands per exposure group with three methods of reporting values <LOQ (best estimate, ½ LOQ and zero). Values are normalized for one day (24h) of use of the silicone band. Detection frequencies can be found in Table S1.

**Chemical** (ng/g of silicone band per day)	**<LOQ = best estimate**		**<LOQ = ½ LOQ**		**<LOQ = zero**	
	**Exposed** (n=41)	**Reference** (n=36)	**Exposed** (n=41)	**Reference** (n=36)	**Exposed** (n=41)	**Reference** (n=36)
**Naphthalene**	68.3 [34.0, 162]	110 [42.6, 168]	68.3 [34.0, 162]	110 [42.6, 168]	68.3 [34.0, 162]	110 [42.6, 168]
**Acenaphthylene**	0.00 [0.00, 6.15]	2.94 [0.00, 12.8]	0.42 [0.25, 6.15]	2.94 [0.24, 12.8]	0.00 [0.00, 6.15]	2.94 [0.00, 12.8]
**Acenaphthene**	1.79 [0.00, 13.0]	1.64 [0.00, 5.05]	1.79 [0.15, 13.0]	1.64 [0.14, 5.05]	1.79 [0.00, 13.0]	1.64 [0.00, 5.05]
**Fluorene**	12.2 [0.00, 21.3]	0.00 [0.00, 8.24]	12.2 [0.19, 21.3]	0.24 [0.15, 8.24]	12.2 [0.00, 21.3]	0.00 [0.00, 8.24]
**Phenanthrene**	23.2 [11.6, 37.8]	33.5 [13.7, 66.0]	23.2 [11.6, 37.8]	33.5 [13.7, 66.0]	23.2 [11.6, 37.8]	33.5 [13.7, 66.0]
**Anthracene**	1.39 [0.00, 14.9]	0.51 [0.00, 19.9]	1.39 [0.17, 14.9]	0.51 [0.17, 19.9]	1.39 [0.00, 14.9]	0.51 [0.00, 19.9]
**Fluoranthene**	0.00 [0.00, 4.38]	0.00 [0.00, 4.43]	0.14 [0.08, 4.38]	0.10 [0.08, 4.43]	0.00 [0.00, 4.38]	0.00 [0.00, 4.43]
**Pyrene**	6.68 [3.18, 11.5]	2.40 [0.00, 5.60]	6.68 [3.18, 11.5]	2.40 [0.10, 5.60]	6.68 [3.18, 11.5]	2.40 [0.00, 5.60]
**Benz(a)anthracene**	2.15 [0.00, 6.49]	0.00 [0.00, 3.97]	2.15 [0.09, 6.49]	0.13 [0.08, 3.97]	2.15 [0.00, 6.49]	0.00 [0.00, 3.97]
**Chrysene**	0.00 [0.00, 0.00]	0.00 [0.00, 0.92]	0.06 [0.06, 0.10]	0.06 [0.06, 0.92]	0.00 [0.00, 0.00]	0.00 [0.00, 0.92]
**Benzo(b+k)fluoranthene**	6.85 [0.00, 30.74]	0.00 [0.00, 34.33]	6.85 [0.16, 30.74]	0.23 [0.15, 34.33]	6.85 [0.00, 30.74]	0.00 [0.00, 34.33]
**Benzo(a)pyrene**	0.00 [0.00, 42.6]	5.02 [0.00, 40.2]	0.29 [0.20, 42.6]	5.02 [0.20, 40.2]	0.00 [0.00, 42.6]	5.02 [0.00, 40.2]
**Dibenz(a,h)antthracene**	0.00 [0.00, 0.00]	0.00 [0.00, 4.21])	0.11 [0.10, 0.18]	0.11 [0.10, 4.34]	0.00 [0.00, 0.00]	0.00 [0.00, 4.21]
**Benzo(ghi)perylene**	11.1 [0.00, 69.1]	0.00 [0.00, 66.3]	11.1 [0.45, 69.1]	0.65 [0.43, 66.3]	11.1 [0.00, 69.1]	0.00 [0.00, 66.3]
**Indeno(1,2,3-cd)pyrene**	5.37 [0.00, 16.0]	0.00 [0.00, 21.4]	5.37 [0.12, 16.0]	0.14 [0.11, 21.4]	5.37 [0.00, 16.0]	0.00 [0.00, 21.4]
**ΣPAH**	291 [113, 515]	288 [124, 590]	292 [116, 515]	289 [126, 591]	291 [113, 515]	288 [124, 590]
**TPHP**	41.5 [0.00, 364]	0.00 [0.00, 35.0]	41.5 [1.72, 364]	2.26 [1.65, 35.0]	41.5 [0.00, 364]	0.00 [0.00, 35.0]
**TMPP**	197 [0.00, 1108]	210 [0.00, 928]	197 [1.89, 1108]	210 [1.87, 928]	197 [0.00, 1108]	210 [0.00, 928]
**o-TMPP**	0.00 [0.00, 7.87]	0.00 [0.00, 4.27]	2.20 [1.76, 7.87]	1.76 [1.67, 4.27]	0.00 [0.00, 7.87]	0.00 [0.00, 4.27]
**TNBP**	0.00 [0.00, 22.6]	0.00 [0.00, 0.28]	3.16 [2.81, 22.6]	2.81 [2.65, 4.01]	0.00 [0.00, 22.6]	0.00 [0.00, 0.00]
**TCEP**	0.00 [0.00, 5.84]	0.00 [0.00, 12.2]	3.27 [3.03, 5.84]	4.80 [3.02, 12.2]	0.00 [0.00, 5.84]	0.00 [0.00, 12.2]
**TCIPP**	84.7 [0.00, 160]	28.6 [0.00, 112]	84.7 [3.57, 160]	28.6 [4.68, 112]	84.7 [0.00, 160]	28.6 [0.00, 112]
**TDCIPP**	0.00 [0.00, 57.6]	0.00 [0.00, 15.6]	1.55 [1.38, 57.64]	1.56 [1.31, 15.6]	0.00 [0.00, 57.6]	0.00 [0.00, 15.6]
**ΣOPE**	871.88 [74.9, 1780.8]	360.75 [119.3, 1128]	877.56 [87.1, 1789]	367.10 [130.6, 1133]	871.88 [74.9, 1780.8]	359.49 [119.3, 1128]

IQR, inter-quartile range (25th-75th); LOQ, limit of quantification; OPE, organophosphate esters; PAH, polycyclic aromatic hydrocarbons; TPHP, Triphenyl phosphate; TMPPMIX, Tri(methyl phenyl) phosphate mixture of isomers; TMPP, Tris(2-methylphenyl) phosphate; TNBP, Tri-n-butyl phosphate; TCEP, Tris(2-chloroethyl)phosphate; TCIPP, Tris(chloroisopropyl)phosphate; TDCIPP, Tris(1,3-dichloro-2-propyl)phosphate

Table S7 - Median [IQR] levels of individual PAHs and OPEs concentrations per job function in silicone bands. Values are best estimates normalized for one day (24h) of use of the silicone band.

	**Aircraft engineer** (n=14[a)])	**Munition specialist** (n=5)	**Crew chief** (n=17)	**Fuel operator** (n=6)	**Avionics** (n=6)	**Office worker** (n=31[a)])
**Deployed time** (h)	21.8 ± 4.3	20.6 ± 3.2	21.1 ± 3.3	22.5 ± 3.1	22.9 ± 4.6	22.2 ± 4.0
**Naphthalene** (ng/g per day)	45.3 [35.6, 58.0]	34.0 [21.4, 68.3]	89.6 [33.1, 252]	120 [79.1, 164]	120 [51.0, 156]	110 [44.1, 177]
**Acenaphthylene** (ng/g per day)	0.00 [0.00, 3.62]	0.00 [0.00, 18.4]	0.00 [0.00, 11.6]	2.21 [0.00, 5.53]	1.10 [0.00, 19.6]	3.30 [0.00, 12.2]
**Acenaphthene** (ng/g per day)	3.42 [1.79, 13.0]	3.54 [2.57, 3.81]	0.00 [0.00, 14.1]	0.00 [0.00, 0.00]	0.73 [0.00, 2.18]	1.90 [0.00, 5.32]
**Fluorene** (ng/g per day)	7.36 [0.00, 12.2]	0.00 [0.00, 0.00]	15.4 [0.00, 26.3]	32.7 [15.8, 50.4]	0.00 [0.00, 0.00]	2.64 [0.00, 8.81]
**Phenanthrene** (ng/g per day)	18.3 [10.2, 30.1]	14.4 [14.1, 25.4]	29.9 [11.6, 37.8]	38.5 [23.2, 55.4]	13.2 [4.23, 16.4]	42.6 [17.3, 91.1]
**Anthracene** (ng/g per day)	0.50 [0.00, 5.99]	0.00 [0.00, 7.86]	2.22 [0.00, 14.9]	10.7 [0.00, 21.7]	0.00 [0.00, 1.79]	0.73 [0.00, 39.1]
**Fluoranthene** (ng/g per day)	1.70 [0.00, 3.25]	0.00 [0.00, 0.00]	0.00 [0.00, 4.38]	5.07 [3.40, 9.43]	0.00 [0.00, 0.00]	0.00 [0.00, 5.66]
**Pyrene** (ng/g per day)	3.42 [0.00, 7.74]	2.54 [0.00, 6.68]	10.6 [6.80, 12.5]	5.04 [3.89, 6.41]	2.11 [0.00, 4.48]	2.40 [0.00, 6.33]
**Benz(a)anthracene** (ng/g per day)	1.64 [0.00, 8.05]	0.00 [0.00, 0.00]	3.68 [0.00, 19.6]	3.76 [2.64, 5.54]	0.00 [0.00, 0.00]	0.27 [0.00, 4.42]
**Chrysene** (ng/g per day)	0.00 [0.00, 0.00]	0.00 [0.00, 0.00]	0.00 [0.00, 0.00]	2.11 [0.32, 3.65]	0.00 [0.00, 0.00]	0.00 [0.00, 2.25]
**Benzo(b+k)fluoranthene (ng/g.day)**	6.85 [0.00, 30.5]	0.00 [0.00, 0.00]	5.65 [0.00, 27.8]	34.1 [22.7, 51.7]	0.00 [0.00, 0.00]	5.40 [0.00, 44.0]
**Benzo(a)pyrene** (ng/g per day)	0.00 [0.00, 53.2]	0.00 [0.00, 0.00]	0 [0.00, 42.55]	24.25 [4.24, 55.99]	2.33 [0.00, 5.20]	14.46 [0.00, 53.83]
**Dibenz(a,h)anthracene** (ng/g per day)	0.00 [0.00, 0.00]	0.00 [0.00, 0.00]	0.00 [0.00, 0.00]	0.00 [0.00, 8.87]	0.00 [0.00, 0.00]	0.00 [0.00, 25.5]
**Benzo(ghi)perylene** (ng/g per day)	11.1 [0.00, 69.1]	0.00 [0.00, 0.00]	0.00 [0.00, 74.7]	51.9 [40.7, 97.1]	0.00 [0.00, 0.00]	10.4 [0.00, 75.2]
**Indeno(1,2,3-cd)pyrene** (ng/g per day)	10.2 [0.00, 21.9]	0.00 [0.00, 0.64]	1.89 [0.00, 15.5]	11.3 [5.09, 44.7]	5.81 [0.00, 34.7]	0.00 [0.00, 16.4]
**ΣPAH** (ng/g per day)	154 [121, 492]	100 [90.7, 146]	425 [113, 532]	336 [278, 541]	181 [109, 299]	291 [127, 620]
**TPHP** (ng/g per day)	0.00 [0.00, 41.5]	0.00 [0.00, 63.9]	434 [92.0, 572]	0.00 [0.00, 72.5]	0.00 [0.00, 0.00]	0.00 [0.00, 51.8]
**TMPP** (ng/g per day)	243 [0.00, 983]	717 [26.2, 1469]	683 [0.00, 1787]	23.9 [0.00, 160]	747 [248, 1866]	145 [0.00, 756]
**o-TMPP** (ng/g per day)	0.00 [0.00, 11.3]	5.72 [0.00, 6.72]	0.00 [0.00, 7.87]	0.00 [0.00, 0.00]	3.02 [0.70, 10.1]	0.00 [0.00, 3.11]
**TNBP** (ng/g per day)	7.00 [0.00, 22.6]	0.00 [0.00, 91.3]	0.00 [0.00, 108]	0.00 [0.00, 4.94]	8.95 [0.00, 66.2]	0.00 [0.00, 0.00]
**TCEP** (ng/g per day)	0.00 [0.00, 6.06]	0.00 [0.00, 0.00]	0.00 [0.00, 5.34]	17.1 [0.00, 34.6]	0.00 [0.00, 0.00]	7.15 [0.00, 12.2]
**TCIPP** (ng/g per day)	71.7 [0.00, 147]	55.6 [0.00, 107]	102 [56.2, 222]	75.7 [16.5, 129]	25.9 [15.7, 31.1]	36.7 [0.00, 158]
**TDCIPP** (ng/g per day)	0.00 [0.00, 0.00]	0 [0, 0]	0 [0.00, 247.45]	12.00 [0.00, 47.68]	0 [0.00, 6.01]	0 [0.00, 17.57]
**ΣOPE** (ng/g per day)	407 [11.8, 1188]	830 [81.8, 1737]	1411 [503, 3419]	187 [49.7, 426]	826 [302, 1986]	340 [76.3, 820]

IQR, inter-quartile range (25th-75th); LOQ, limit of quantification; OPE, organophosphate esters; PAH, polycyclic aromatic hydrocarbons; TPHP, Triphenyl phosphate; TMPPMIX, Tri(methyl phenyl) phosphate mixture of isomers; TMPP, Tris(2-methylphenyl) phosphate; TNBP, Tri-n-butyl phosphate; TCEP, Tris(2-chloroethyl)phosphate; TCIPP, Tris(chloroisopropyl)phosphate; TDCIPP, Tris(1,3-dichloro-2-propyl)phosphate; [a)] One lost wristband sample, the final sample size is n=14 aircraft eng. and n=30 office workers, respectively.

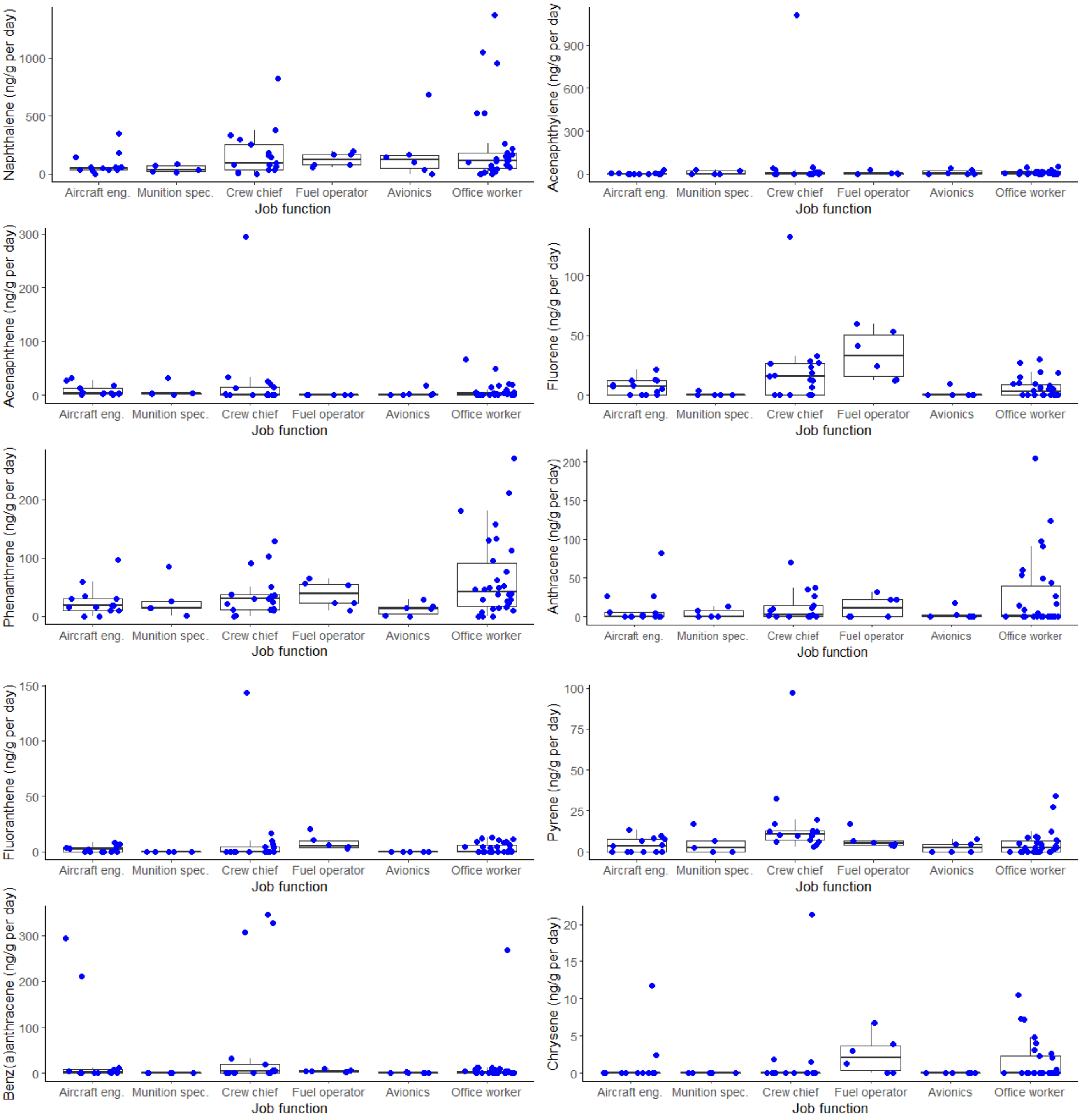

Figure S5 – Levels of individual polycyclic aromatic hydrocarbons (PAH) in silicone bands (normalized for 24h) per job function (n=77), best estimates

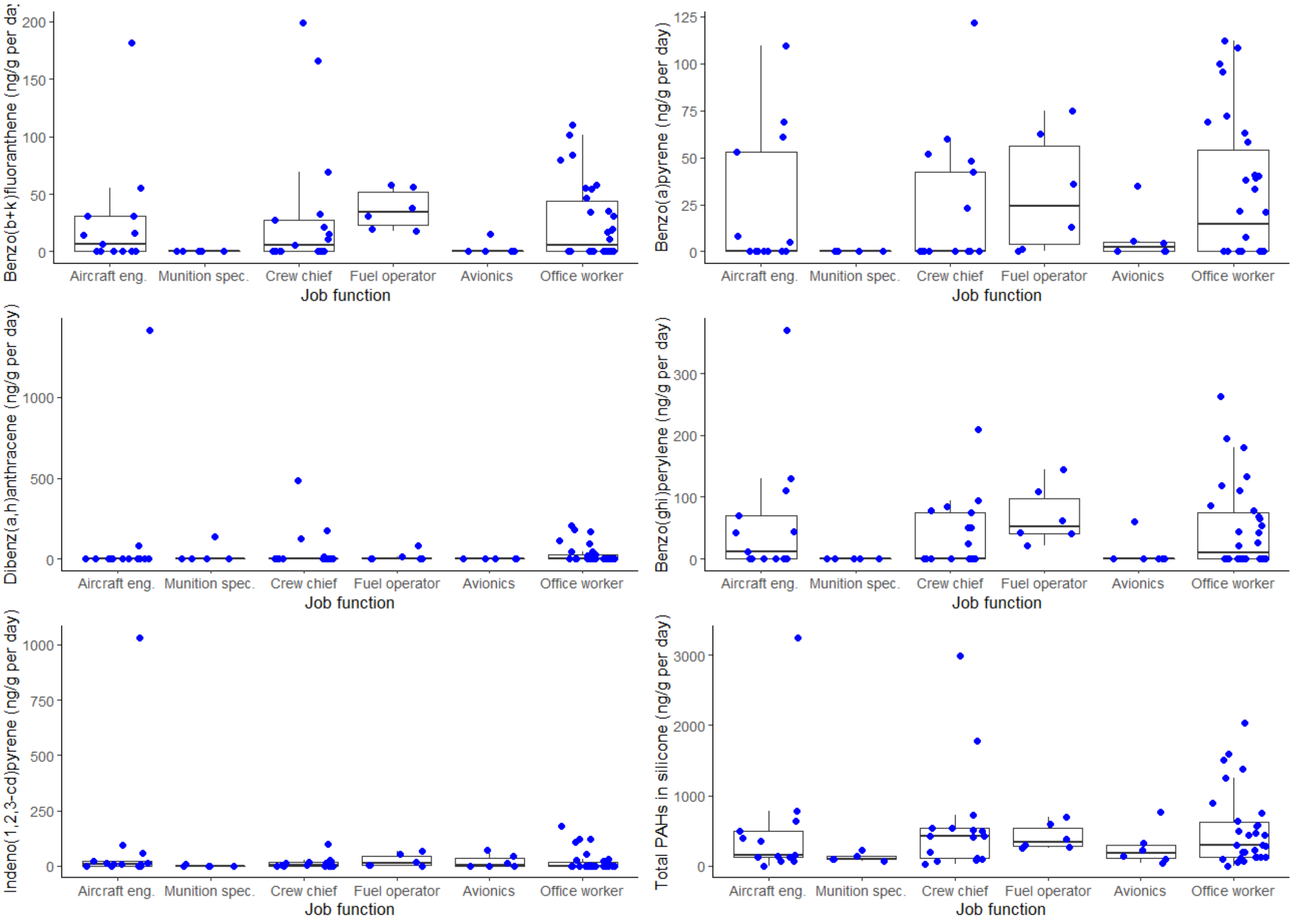

Figure S5 (cont) – Levels of individual and total polycyclic aromatic hydrocarbons (PAH) in silicone bands (normalized for 24h) per job function (n=77), best estimates

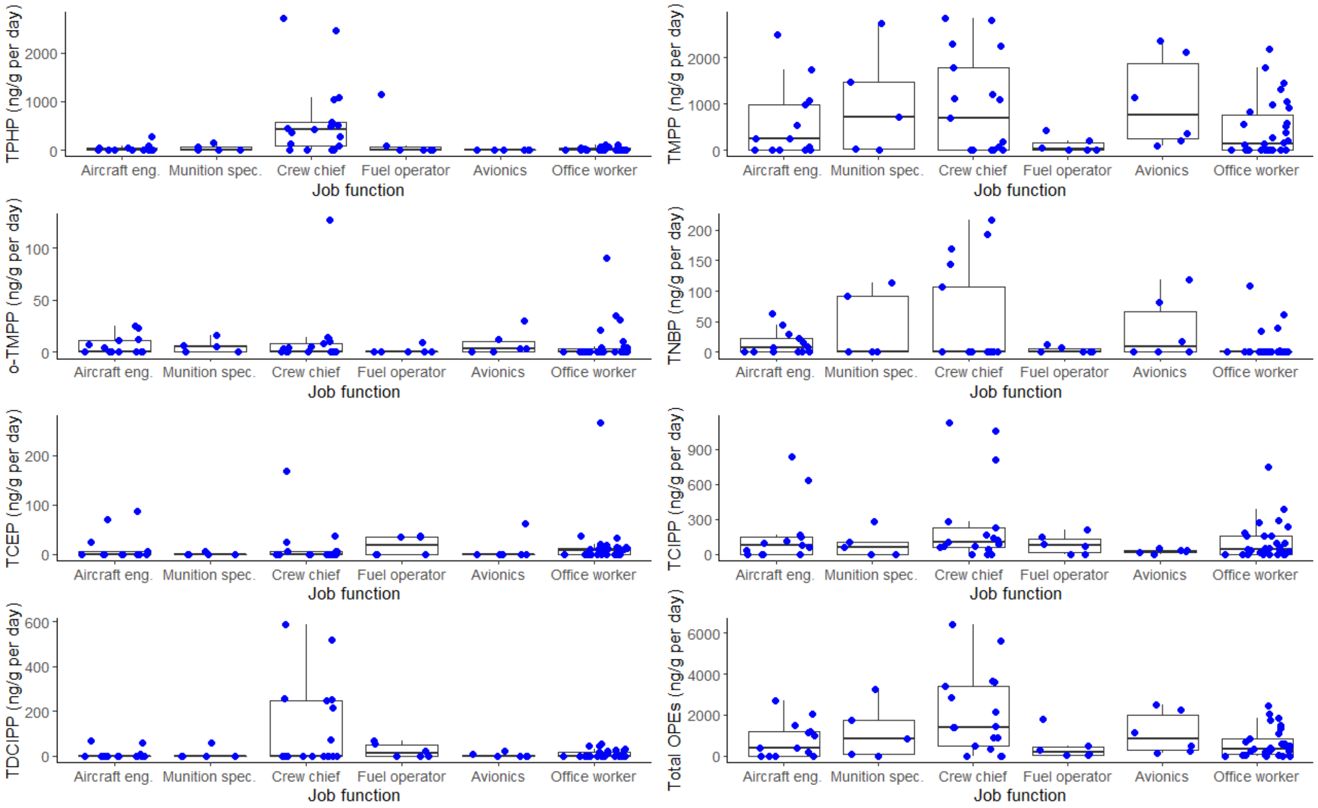

Figure S6 – Levels of individual and total organophosphate esters (OPE) in silicone bands (normalized for 24h) per job function (n=77), values are best estimates. TPHP, Triphenyl phosphate; TMPPMIX, Tri(methyl phenyl) phosphate mixture of isomers; TMPP, Tris(2-methylphenyl) phosphate; TNBP, Tri-n-butyl phosphate; TCEP, Tris(2-chloroethyl)phosphate; TCIPP, Tris(chloroisopropyl)phosphate; TDCIPP, Tris(1,3-dichloro-2-propyl)phosphate

Table S8 – Median [IQR] levels of individual and total PAHs on skin wipes per exposure group with three methods of reporting values <LOQ (best estimate, ½ LOQ and zero). Values are normalized for 1h (to correct for the different sampling timings). Detection frequencies can be found in Table S1.

**Chemical** (ng/cm² per 1h)	**<LOQ = best estimate**		**<LOQ = ½ LOQ**		**<LOQ = zero**	
	**Exposed** (n=27)	**Reference** (n=27)	**Exposed** (n=27)	**Reference** (n=27)	**Exposed** (n=41)	**Reference** (n=36)
**Naphthalene**	0.00 [0.00, 0.87]	0.00 [0.00, 0.00]	0.03 [0.02, 0.04]	0.02 [0.02, 0.04]	0.00 [0.00, 0.00]	0.00 [0.00, 0.00]
**Acenaphthylene**	0.01 [0.00, 0.14]	0.08 [0.00, 0.21]	0.11 [0.08, 0.14]	0.14 [0.07, 0.24]	0.00 [0.00, 0.07]	0.00 [0.00, 0.19]
**Acenaphthene**	0.00 [0.00, 0.00]	0.00 [0.00, 0.00]	0.05 [0.04, 0.06]	0.05 [0.03, 0.08]	0.00 [0.00, 0.00]	0.00 [0.00, 0.00]
**Fluorene**	0.00 [0.00, 0.00]	0.00 [0.00, 0.00]	0.06 [0.04, 0.07]	0.05 [0.04, 0.08]	0.00 [0.00, 0.00]	0.00 [0.00, 0.00]
**Phenanthrene**	0.00 [0.00, 0.00]	0.00 [0.00, 0.00]	0.05 [0.04, 0.07]	0.05 [0.03, 0.07]	0.00 [0.00, 0.00]	0.00 [0.00, 0.00]
**Anthracene**	0.00 [0.00, 0.04]	0.00 [0.00, 0.00]	0.08 [0.05, 0.09]	0.07 [0.04, 0.10]	0.00 [0.00, 0.00]	0.00 [0.00, 0.00]
**Fluoranthene**	0.00 [0.00, 0.00]	0.00 [0.00, 0.02]	0.04 [0.02, 0.04]	0.04 [0.02, 0.05]	0.00 [0.00, 0.00]	0.00 [0.00, 0.02]
**Pyrene**	0.00 [0.00, 0.01]	0.00 [0.00, 0.00]	0.03 [0.03, 0.04]	0.03 [0.02, 0.05]	0.00 [0.00, 0.00]	0.00 [0.00, 0.00]
**Benz(a)anthracene**	0.00 [0.00, 0.06]	0.01 [0.00, 0.07]	0.04 [0.03, 0.08]	0.05 [0.02, 0.10]	0.00 [0.00, 0.06]	0.01 [0.00, 0.07]
**Chrysene**	0.00 [0.00, 0.09]	0.04 [0.00, 0.11]	0.03 [0.02, 0.09]	0.04 [0.03, 0.11]	0.00 [0.00, 0.09]	0.01 [0.00, 0.11]
**Benzo(b+k)fluoranthene**	0.00 [0.00, 0.00]	0.00 [0.00, 0.00]	0.07 [0.05, 0.08]	0.06 [0.04, 0.09]	0.00 [0.00, 0.00]	0.00 [0.00, 0.00]
**Benzo(a)pyrene**	0.00 [0.00, 0.14]	0.15 [0.00, 0.94]	0.11 [0.08, 0.22]	0.19 [0.10, 0.94]	0.00 [0.00, 0.14]	0.15 [0.00, 0.94]
**Dibenz(a,h)antthracene**	0.00 [0.00, 0.40]	0.00 [0.00, 0.11]	0.06 [0.05, 0.43]	0.06 [0.03, 0.15]	0.00 [0.00, 0.40]	0.00 [0.00, 0.11]
**Benzo(ghi)perylene**	0.00 [0.00, 0.00]	0.00 [0.00, 0.00]	0.20 [0.13, 0.22]	0.14 [0.10, 0.25]	0.00 [0.00, 0.00]	0.00 [0.00, 0.00]
**Indeno(1,2,3-cd)pyrene**	0.00 [0.00, 0.38]	0.08 [0.00, 0.41]	0.06 [0.04, 0.42]	0.12 [0.05, 0.41]	0.00 [0.00, 0.38]	0.07 [0.00, 0.41]
**ΣPAH**	0.63 [0.12, 2.53]	0.92 [0.15, 1.95]	1.50 [0.98, 3.21]	1.40 [0.98, 2.55]	0.58 [0.03, 2.51]	0.92 [0.10, 1.93]

IQR, inter-quartile range (25th-75th); LOQ, limit of quantification; PAH, polycyclic aromatic hydrocarbons;

Table S9 – Median [IQR] levels of individual urinary metabolites of PAHs per exposure group with three methods of reporting values <LOQ (best estimate, ½ LOQ and zero). Detection frequencies can be found in Table S1.

**Chemical** (µmol/mol creatinine)	**<LOQ = best estimate**		**<LOQ = ½ LOQ**		**<LOQ = zero**	
	**Exposed** (n=41)	**Reference** (n=37)	**Exposed** (n=41)	**Reference** (n=37)	**Exposed** (n=41)	**Reference** (n=37)
**1-NAP**	0.48 [0.00, 0.88]	0.53 [0.00, 1.64]	0.48 [0.11, 0.88]	0.53 [0.11, 1.64]	0.48 [0.00, 0.88]	0.53 [0.00, 1.64]
**2-NAP**	2.07 [1.44, 3.22]	2.91 [1.78, 3.87]	2.07 [1.44, 3.22]	2.91 [1.78, 3.87]	2.07 [1.44, 3.22]	2.91 [1.78, 3.87]
**2-FLU**	0.11 [0.08, 0.13]	0.10 [0.07, 0.14]	0.11 [0.08, 0.13]	0.10 [0.07, 0.14]	0.11 [0.08, 0.13]	0.10 [0.07, 0.14]
**1-PHE**	0.06 [0.03, 0.08]	0.07 [0.04, 0.10]	0.06 [0.03, 0.08]	0.07 [0.04, 0.10]	0.06 [0.03, 0.08]	0.07 [0.03, 0.10]
**2,3-PHE**	0.04 [0.03, 0.06]	0.05 [0.03, 0.07]	0.04 [0.03, 0.05]	0.05 [0.03, 0.07]	0.04 [0.00, 0.05]	0.04 [0.00, 0.07]
**4-PHE**	0.00 [0.00, 0.00]	0.00 [0.00, 0.01]	0.02 [0.01, 0.02]	0.02 [0.01, 0.03]	0.00 [0.00, 0.00]	0.00 [0.00, 0.00]
**1-PYR**	0.05 [0.03, 0.06]	0.04 [0.03, 0.07]	0.05 [0.03, 0.06]	0.04 [0.03, 0.07]	0.04 [0.02, 0.06]	0.04 [0.00, 0.07]
**ΣOH-PAH**	2.91 [2.11, 4.30]	4.16 [2.94, 5.19]	2.92 [2.12, 4.31]	4.16 [2.95, 5.26]	2.86 [2.07, 4.25]	4.11 [2.89, 5.09]

IQR, inter-quartile range (25th-75th); LOQ, limit of quantification; 1-NAP, 1-hydroxynapthalene; 2-NAP, 2-hydroxynapthalene; 2-FLU, 2-hydroxyfluorene; 1,2,3,4-PHE, 1,2,3,4-hydroxyphenanthrene; 1-PYR, 1-hydroxypyrene. The arithmetic average levels of creatinine were 11.17±5.47 mmol/L for non-exposed subjects and 12.23±5.55 mmol/L for exposed subjects.

Table S10 - Median [IQR] levels of individual urinary metabolites of PAHs per job function and excluding females. Values are best estimates

Urine	**Aircraft engineer** (n=14)[a)]	**Munition specialist** (n=5)[a)]	**Crew chief** (n=17)	**Fuel operator** (n=5)	**Avionics** (n=6)	**Office worker** (n=23)
**Creatinine** (mmol/L)	11.6 [8.64, 12.2]	13.8 [8.23, 20.7]	10.7 [6.68, 15.5]	11.3 [10.78, 15.38]	12.9 [11.8, 16.6]	9.20 [6.34, 16.2]
**1-NAP** (µmol/mol crea)	0.00 [0.00, 0.58]	0.42 [0.13, 0.82]	0.57 [0.00, 0.96]	0.79 [0.64, 1.08]	0.98 [0.39, 1.49]	0.53 [0.00, 1.65]
**2-NAP** (µmol/mol crea)	1.78 [1.56, 2.35]	2.51 [2.06, 2.73]	2.07 [1.44, 3.25]	3.22 [2.15, 3.27]	1.72 [1.40, 2.17]	2.91 [1.83, 3.61]
**2-FLU** (µmol/mol crea)	0.10 [0.08, 0.13]	0.08 [0.06, 0.10]	0.12 [0.10, 0.15]	0.12 [0.10, 0.13]	0.09 [0.07, 0.11]	0.10 [0.07, 0.14]
**1-PHE** (µmol/mol crea)	0.06 [0.05, 0.11]	0.05 [0.03, 0.08]	0.06 [0.03, 0.08]	0.08 [0.07, 0.08]	0.04 [0.02, 0.09]	0.07 [0.04, 0.10]
**2,3-PHE** (µmol/mol crea)	0.04 [0.03, 0.07]	0.04 [0.04, 0.05]	0.05 [0.03, 0.05]	0.03 [0.02, 0.04]	0.03 [0.03, 0.06]	0.05 [0.04, 0.07]
**4-PHE** (µmol/mol crea)	0.00 [0.00, 0.00]	0.01 [0.00, 0.01]	0.00 [0.00, 0.00]	0.00 [0.00, 0.02]	0.00 [0.00, 0.01]	0.00 [0.00, 0.02]
**1-PYR** (µmol/mol crea)	0.05 [0.04, 0.08]	0.04 [0.03, 0.04]	0.05 [0.03, 0.06]	0.04 [0.02, 0.09]	0.03 [0.03, 0.05]	0.05 [0.03, 0.07]
**ΣOH-PAH** (µmol/mol crea)	2.79 [1.85, 3.76]	3.55 [2.71, 3.89]	2.90 [2.16, 4.32]	4.37 [3.51, 5.30]	2.70 [2.10, 4.05]	3.96 [2.99, 4.85]

IQR, inter-quartile range (25th-75th); ND, not determined; 1-NAP, 1-hydroxynapthalene; 2-NAP, 2-hydroxynapthalene; 2-FLU, 2-hydroxyfluorene; 1,2,3,4-PHE, 1,2,3,4-hydroxyphenanthrene; 1-PYR, 1-hydroxypyrene. [a)] One lost sample, final n=13 and 4 respectively.

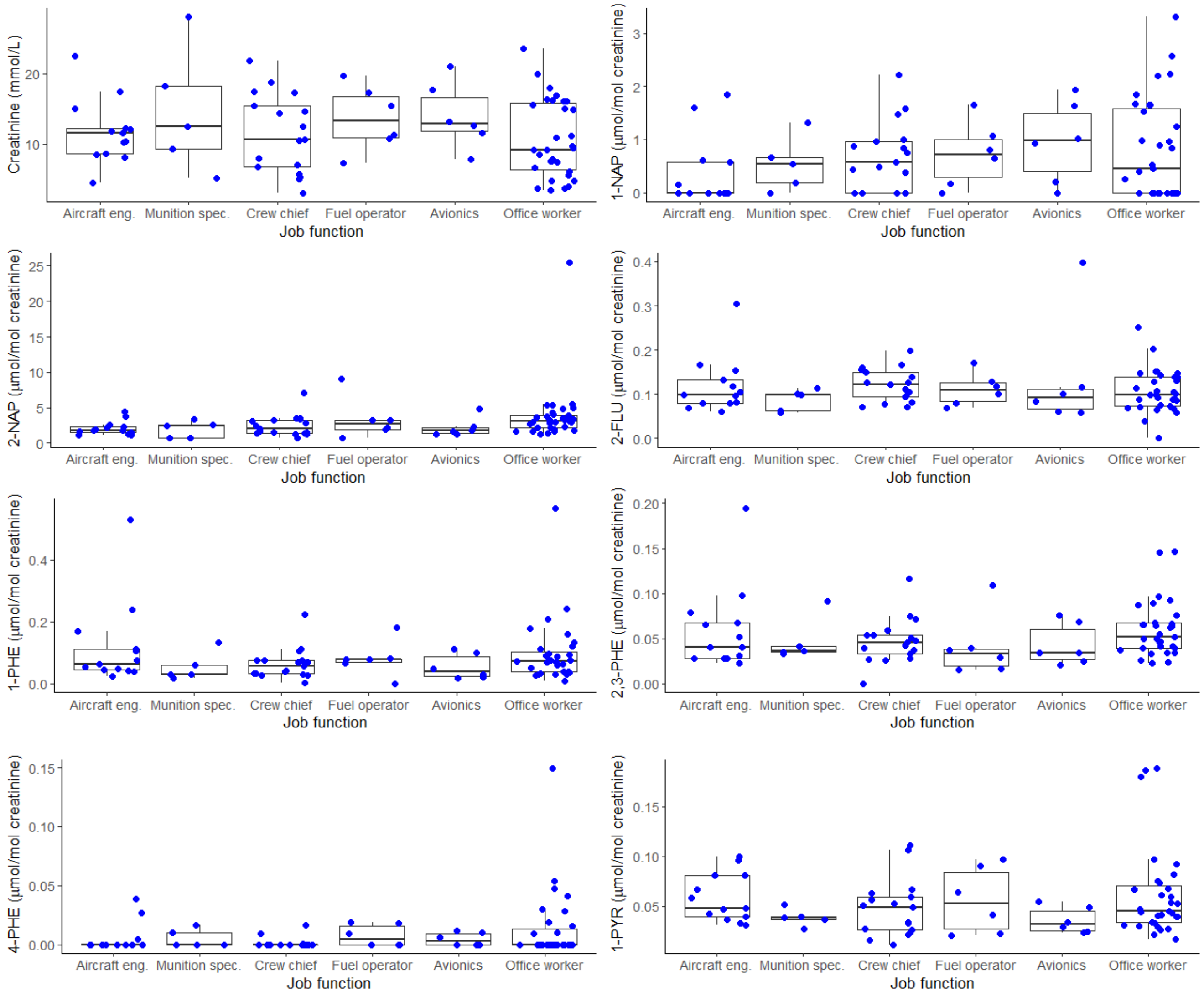

Figure S7 – Levels of urinary creatinine and individual urinary metabolites of polycyclic aromatic hydrocarbons per job function (n=78). Values are best estimates. 1-NAP, 1-hydroxynapthalene; 2-NAP, 2-hydroxynapthalene; 2-FLU, 2-hydroxyfluorene; 1,2,3,4-PHE, 1,2,3,4-hydroxyphenanthrene; 1-PYR, 1-hydroxypyrene.

Table S11 – Median [IQR] levels of individual PAHs and OPEs in silicone bands per campaign day. Values are best estimates adjusted for 24h deployed time.

	**Campaign 1** (n=22)	**Campaign 2** (n=21)	**Campaign 3** (n=19)	**Campaign 4** (n=15)	**Total** (n=77)	**ANOVA test** (log transformed data)
**Deployment time** (h)	23.3 (3.0)	21.4 (3.6)	21.8 (3.9)	20.3 (4.4)	21.8 (3.8)	F=2.3; df1=3; df2=37.4; p=0.091
Job functions						
**Aircraft eng.**	4	4	4	1	13	
**Crew chief**	4	5	4	4	17	
**Fuel operator**	3	3	0	0	6	
**Munition spec.**	0	1	4	0	5	
**Avionics**	1	0	3	2	6	
**Office worker**	10	8	4	8	30	
**Naphthalene** (ng/g per day)	178 [161, 480]	52.8 [18.0, 89.6]	52.0 [34.5, 90.1]	57.0 [12.9, 92.1]	79.1 [35.6, 163]	ND
**Acenaphthylene** (ng/g per day)	4.8 [3.00, 10.4]	0.00 [0.00, 2.60]	0.00 [0.00, 25.6]	0.00 [0.00, 5.60]	2.20 [0.00, 11.2]	ND
**Acenaphthene** (ng/g per day)	0.00 [0.00, 2.80]	0.00 [0.00, 21.6]	2.70 [0.60, 3.70]	1.80 [0.00, 4.40]	1.80 [0.00, 5.70]	ND
**Fluorene** (ng/g per day)	12.7 [8.10, 20.8]	7.80 [3.40, 26.9]	0.00 [0.00, 0.00]	0.00 [0.00, 0.00]	5.40 [0.00, 13.0]	ND
**Phenanthene** (ng/g per day)	31.3 [14.4, 53.0]	30.4 [23.6, 84.7]	14.4 [7.10, 29.3]	34.2 [11.1, 44.6]	28.6 [12.4, 51.3]	ND
**Anthracene** (ng/g per day)	29.5 [18.7, 52.4]	0.00 [0.00, 4.20]	0.80 [0.00, 3.50]	0.00 [0.00, 0.00]	0.80 [0.0, 17.8]	ND
**Fluoranthene** (ng/g per day)	5.50 [3.50, 8.00]	3.10 [0.00, 9.30]	0.00 [0.00, 0.00]	0.00 [0.00, 0.00]	0.00 [0.00, 4.40]	ND
**Pyrene** (ng/g per day)	4.80 [3.50, 8.30]	5.20 [0.00, 9.70]	4.20 [0.00, 7.30]	1.40 [0.00, 9.10]	4.40 [0.00, 9.10]	ND
**Benz(a)anthracene** (ng/g per day)	6.30 [3.70, 10.5]	3.40 [0.00, 2112]	0.00 [0.00, 0.00]	0.00 [0.00, 0.00]	0.00 [0.00, 6.00]	ND
**Chrysene** (ng/g per day)	2.40 [0.20, 6.30]	0.00 [0.00, 0.00]	0.00 [0.00, 0.00]	0.00 [0.00, 0.00]	0.00 [0.00, 0.10]	ND
**Benzo(b+k)fluoranthene** (ng/g per day)	50.5 [28.5, 66.6]	15.2 [5.60, 31.1]	0.00 [0.00, 0.00]	0.00 [0.00, 0.00]	0.00 [0.00, 31.2]	ND
**Benzo(a)pyrene** (ng/g per day)	59.7 [41.3, 74.0]	0.00 [0.00, 12.8]	0.00 [0.00, 0.00]	0.00 [0.00, 0.00]	0.00 [0.00, 40.9]	ND
**Dibenz(a,h)anthracene** (ng/g per day)	13.2 [0.00, 72.9]	0.00 [0.00, 94.3]	0.00 [0.00, 0.00]	0.00 [0.00, 0.00]	0.00 [0.00, 0.00]	ND
**Benzo(ghi)perylene** (ng/g per day)	110 [70.5, 141]	25.8 [0.00, 44.2]	0.00 [0.00, 0.00]	0.00 [0.00, 0.00]	0.00 [0.00, 69.0]	ND
**Indeno(1,2,3-cd) pyrene** (ng/g per day)	8.60 [0.00, 56.1]	5.00 [0.00, 17.3]	1.90 [0.00, 12.3]	0.00 [0.00, 0.00]	0.60 [0.00, 17.3]	ND
**Total PAHs** (ng/g per day)	615 [449, 1343]	348 [232, 515]	121 [92.0, 206]	126 [68.7, 167]	291 [120, 545]	F=29.0; df1=3; df2=37.4; **p<0.0001**[a)]
**TPHP** (ng/g per day)	0.00 [0.00, 38.8]	0.00 [0.00, 65.5]	0.00 [0.00, 78.0]	52.9 [0.00, 276]	0.00 [0.00, 92.0]	ND
**TMPP** (ng/g per day)	81.6 [0.00, 349]	0.00 [0.00, 0.00]	983 [298, 1334]	1140 [632, 1778]	209 [0.00, 1077]	ND
**o-TMPP** (ng/g per day)	0.00 [0.00, 0.00]	0.00 [0.00, 5.60]	3.50 [0.00, 10.7]	2.80 [0.00, 22.1]	0.00 [0.00, 6.70]	ND
**TNBP** (ng/g per day)	0.00 [0.00, 0.00]	0.00 [0.00, 0.00]	28.8 [0.00, 99.5]	0.00 [0.00, 47.8]	0.00 [0.00, 12.9]	ND
**TCEP** (ng/g per day)	10.9 [4.70, 34.0]	0.00 [0.00, 0.00]	0.00 [0.00, 0.00]	7.50 [0, 17]	0.00 [0.00, 11.9]	ND
**TCIPP** (ng/g per day)	109 [47.8, 203]	0.00 [0.00, 47.2]	54.4 [26.0, 110]	96.5 [18.3, 258]	55.6 [0.00, 157]	ND
**TDCIPP** (ng/g per day)	25.7 [0.00, 59.6]	0.00 [0.00, 0.00]	0.00 [0.00, 0.00]	0.00 [0.00, 16.6]	0.00 [0.00, 22.2]	
**Total OPEs** (ng/g per day)	437 [185, 968.6]	14.2 [2.80, 298]	1156 [438, 1946]	1505 [880, 2578]	492 [81.8, 1473]	F=9.6; df1=3; df2=35.6; **p<0.0001**[b)]

IQR, inter-quartile range (25th-75th); LOQ, limit of quantification; ND, not determined; OPE, organophosphate esters; PAH, polycyclic aromatic hydrocarbons; TPHP, Triphenyl phosphate; TMPPMIX, Tri(methyl phenyl) phosphate mixture of isomers; TMPP, Tris(2-methylphenyl) phosphate; TNBP, Tri-n-butyl phosphate; TCEP, Tris(2-chloroethyl)phosphate; TCIPP, Tris(chloroisopropyl)phosphate; TDCIPP, Tris(1,3-dichloro-2-propyl)phosphate; [a)] 2 eliminated values (zeros); [b)] 5 eliminated values (zeros)

Table S12– Median [IQR] levels of individual urinary PAHs metabolite concentrations per campaign day. Values are best estimates.

	**Campaign 1** (n=24)	**Campaign 2** (n=20)	**Campaign 3** (n=19)	**Campaign 4** (n=15)	**Total** (n=78)	**ANOVA test** (log transformed data)
Job functions						
**Aircraft eng.**	5	4	4	1	14	-
**Crew chief**	4	5	4	4	17	
**Fuel operator**	3	3	0	0	6	
**Munition spec.**	0	1	4	0	5	
**Avionics**	1	0	3	2	6	
**Office worker**	11	7	4	8	30	
**Creatinine** (mmol/L)	10.6 [6.52, 15.0]	11.2 [6.70, 17.6]	10.2 [7.86, 12.3]	14.6 [10.0, 16.3]	11.0 [7.54, 16.0]	F=1.31; df1=3; df2=39.8; p=0.283
**1-NAP** (µmol/mol crea)	0.90 [0.00, 1.64]	0.46 [0.11, 0.90]	0.54 [0.00, 1.10]	0.00 [0.00, 0.75]	0.50 [0.00, 1.06]	ND
**2-NAP** (µmol/mol crea)	2.35 [1.65, 3.34]	1.92 [1.42, 3.33]	2.29 [1.58, 3.22]	2.77 [1.84, 4.13]	2.39 [1.61, 3.36]	F=0.86; df1=3; df2=38.0; p=0.470
**2-FLU** (µmol/mol crea)	0.12 [0.10, 0.14]	0.10 [0.08, 0.11]	0.07 [0.07, 0.11]	0.11 [0.08, 0.14]	0.10 [0.08, 0.14]	F=1.11; df1=3; df2=35.9; p=0.357
**1-PHE** (µmol/mol crea)	0.10 [0.07, 0.17]	0.06 [0.04, 0.07]	0.04 [0.03, 0.07]	0.06 [0.04, 0.09]	0.07 [0.04, 0.10]	F=4.75; df1=3; df2=37.1; **p=0.007**
**2,3-PHE** (µmol/mol crea)	0.07 [0.04, 0.08]	0.05 [0.04, 0.06]	0.03 [0.03, 0.04]	0.04 [0.03, 0.07]	0.04 [0.03, 0.07]	F=3.12; df1=3; df2=38.0; **p=0.03**
**4-PHE** (µmol/mol crea)	0.00 [0.00, 0.02]	0.00 [0.00, 0.00]	0.00 [0.00, 0.00]	0.00 [0.00, 0.01]	0.00 [0.00, 0.01]	ND
**1-PYR** (µmol/mol crea)	0.071 [0.05, 0.09]	0.03 [0.02, 0.05]	0.04 [0.03, 0.05]	0.05 [0.04, 0.06]	0.05 [0.03, 0.067]	F=6.86; df1=3; df2=38.2; **p<0.001**
**ΣOH-PAH** (µmol/mol crea)	3.77 [3.04, 4.52]	3.33 [2.15, 4.36]	2.91 [2.18, 4.40]	3.66 [2.90, 4.79]	3.63 [2.43, 4.50]	F=0.83; df1=3; df2=39.0; p=0.485

IQR, inter-quartile range (25th-75th); ND, not determined; PAHs, polycyclic aromatic hydrocarbons; 1-NAP, 1-hydroxynapthalene; 2-NAP, 2-hydroxynapthalene; 2-FLU, 2-hydroxyfluorene; 1,2,3,4-PHE, 1,2,3,4-hydroxyphenanthrene; 1-PYR, 1-hydroxypyrene; ΣOH-PAH, total monohydroxylated metabolites of PAHs.

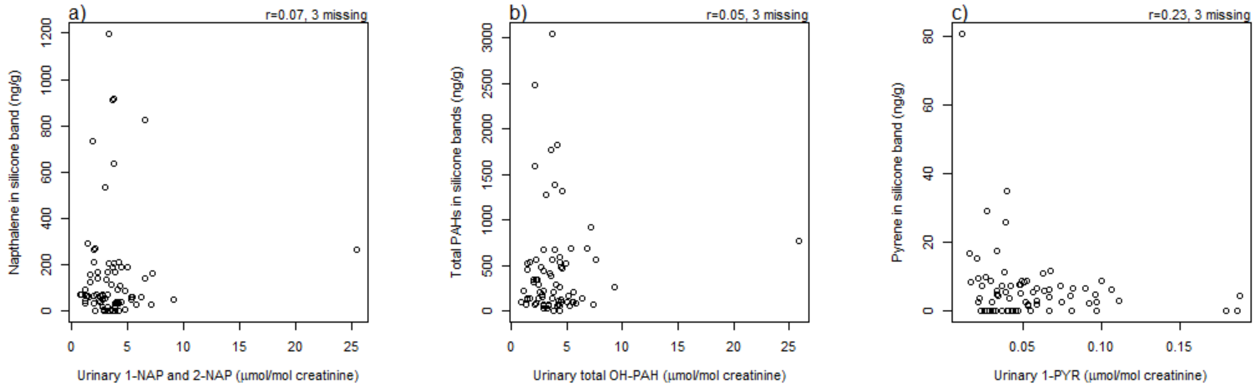

Figure S8 – Correlations between PAHs exposure markers: **a)** silicone band and urinary markers of naphthalene; **b)** total PAHs in silicone bands and urinary OH-PAHs; **c)** silicone band and urinary marker of pyrene; PAHs, polycyclic aromatic hydrocarbons; 1-NAP, 1-hydroxynapthalene; 2-NAP, 2-hydroxynapthalene; 1-PYR, 1-hydroxypyrene; OH-PAH, monohydroxylated metabolites of PAHs.

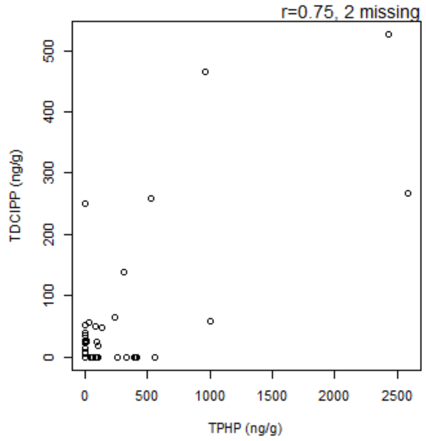

Figure S9 – Correlation between two OPEs (TDCIPP and TPHP) in silicone bands.
Values are not normalized for time of deployment. OPE, organophosphate esters; TPHP, Triphenyl phosphate; TDCIPP, Tris(1,3-dichloro-2-propyl)phosphate;

Table S13 – Average (±SD) of the biomarker levels between the defined reference and exposure groups among male volunteers and p-value from analysis of variance test (females excluded from analysis).

Biomarker	Exposed group	N=42	Reference group	N=37	p-value [a)]
**Total urinary OH-PAHs** (µmol/mol creatinine)	3.39 (±1.7)	39	4.68 (±4.4)	29	0.094
**DNA strand breaks** (number of lesions/10^6 bp)	0.09 (±0.04)	40	0.11 ± (0.04)	27	0.053
**Micronucleated reticulocytes** (%)	3.04 (±1.24)	36	3.1 (±1.45)	26	0.823
**CRP** (mg/L)	1.47 (±2.5)	33	1.38 (±1.5)	24	0.540
**SAA** (mg/L)	14.38 (±15.5)	33	18.55 (±21.1)	24	0.296
**FEV1** (% of predicted)	103 (±14)	32	105 (±13)	25	0.657
**FVC** % of predicted**)**	105 (±12)	32	106 (±11)	25	0.695
**PEF** (% of predicted)	120 (±17)	32	121 (±19)	25	0.807

SD, standard deviation; CRP, C-reactive protein; FEV1, forced expiratory volume in 1 second; FVC, forced vital capacity; OH- PAHs, monohydroxylated metabolites of PAHs; OPE, organophosphate esters; PAHs, polycyclic aromatic hydrocarbons; PEF, peak expiratory flow; SAA, serum amyloid A; [a)] The p-values were determined by the Welch one-way test on normal data (transformed when needed, as reported in statistics description);

Table S14 – Average (±SD) of the biomarker levels between the defined reference and exposure groups without Avionics and 4 Office workers with office in the hangar (separated by glass door). The p-value was determined from analysis of variance test.

Biomarker	Exposed group	N=42	Reference group	N=27	p-value [a)]
**Silicone bands total PAHs** (ng/g per day)	479 (±683)	41	458 (±501)	27	0.778
**Silicone bands total OPEs** (ng/g per day)	1311 (±1552)	41	612 (±697)	27	0.102
**Skin wipes total PAHs** (ng/cm^2 per 1h)	2.05 (±3.02)	27	2.33 (±5.98)	21	0.775
**Total urinary OH-PAHs** (µmol/mol creatinine)	3.29 (±1.7)	41	5.02 (±4.4)	27	**0.003**
**DNA strand breaks** (number of lesions/10^6 bp)	0.09 (±0.04)	42	0.10 (±0.04)	27	0.538
**Micronucleated reticulocytes** (%)	3.01 (±1.2)	37	2.84 (±1.17)	23	0.581
**CRP** (mg/L)	1.96 (±3.9)	35	1.47 (±1.6)	22	0.605
**SAA** (mg/L)	17.84 (±23.9)	35	22.91 (±21.9)	22	0.139
**FEV1** (% of predicted)	103 (±13)	34	107 (±15)	23	0.300
**FVC** (% of predicted)	105 (±12)	34	110 (±15)	23	0.183
**PEF** (% of predicted)	119 (±17)	34	120 (±22)	23	0.900

SD, standard deviation; CRP, C-reactive protein; FEV1, forced expiratory volume in 1 second; FVC, forced vital capacity; OH- PAHs, monohydroxylated metabolites of PAHs; OPE, organophosphate esters; PAHs, polycyclic aromatic hydrocarbons; PEF, peak expiratory flow; SAA, serum amyloid A; [a)] The p-values were determined by the Welch one-way test on normal data (transformed when needed, as reported in statistics description); [b)] Skin wipe data corresponds to the sum of left, right and neck wipes normalized for 1 h;

Table S15 – Regression analysis for biomarkers of effect, using exposure group, age, sex, BMI and relevant health history as predictors (excluding Avionics and 4 office workers)

Explained variable	N=69	R^2	Model p-value	Predictors	Parameter estimate	SE	p-value
**DNA strand breaks**	69	0.031	0.846	Exposure group	-0.174	0.291	0.552
				Age	0.001	0.013	0.954
				Sex (Male)	0.069	0.421	0.870
				BMI	0.032	0.041	0.439
				Health history (diabetes, stroke, cancer)	0.474	0.601	0.433
				Intercept	1.669	1.277	0.196
**Natural logarithm of micronuclei reticulocytes**	60	0.012	0.985	Exposure group	0.045	0.113	0.694
				Age	0.001	0.005	0.904
				Sex (Male)	0.076	0.172	0.660
				BMI	0.006	0.015	0.676
				Health history (diabetes, stroke, cancer)	-0.035	0.216	0.871
				Intercept	0.711	0.493	0.154
**Natural logarithm of CRP**	56	0.277	0.005	Exposure group	-0.098	0.270	0.718
				Age	-0.020	0.013	0.123
				Sex (Male)	-0.280	0.377	0.461
				BMI	0.143	0.035	**0.0002**
				Health history (diabetes and eczema)	-0.184	0.404	0.652
				Intercept	-2.746	1.152	0.021
**Natural logarithm of SAA**	56	0.312	0.002	Exposure group	-0.120	0.247	0.629
				Age	-0.006	0.012	0.612
				Sex (Male)	-1.056	0.344	**0.003**
				BMI	0.107	0.032	**0.002**
				Health history (diabetes and eczema)	-0.517	0.369	0.168
				Intercept	0.916	1.051	0.388
**FEV1**	56	0.050	0.613	Exposure group	-3.547	3.980	0.377
				BMI	-0.642	0.536	0.236
				Health history (asthma)	2.145	14.88	0.886
				Smoke history (occasional or former smoker)	2.513	3.954	0.528
				Intercept	123.69	15.04	<0.0001
**FVC**	56	0.342	0.002	Exposure group	-4.340	3.643	0.239
				BMI	-0.456	0.490	0.357
				Health history (asthma)	3.672	13.62	0.789
				Smoke history (occasional or former smoker)	2.273	3.619	0.533
				Intercept	121.29	13.77	<0.0001
**PEF**	56	0.398	<0.001	Exposure group	1.070	5.209	0.838
				BMI	-0.229	0.701	0.745
				Health history (asthma)	29.99	19.48	0.130
				Smoke history (occasional or former smoker)	4.301	5.174	0.410
				Intercept	123.01	19.69	<0.0001

BMI, body mass index; CRP, C-reactive protein; FEV1, forced expiratory volume in 1 second; FVC, forced vital capacity; OH-PAHs, monohydroxylated metabolites of PAHs; OPE, organophosphate esters; PAHs, polycyclic aromatic hydrocarbons; PEF, peak expiratory flow; R^2, proportion of variance explained by the model; SAA, serum amyloid A; SE, standard error.

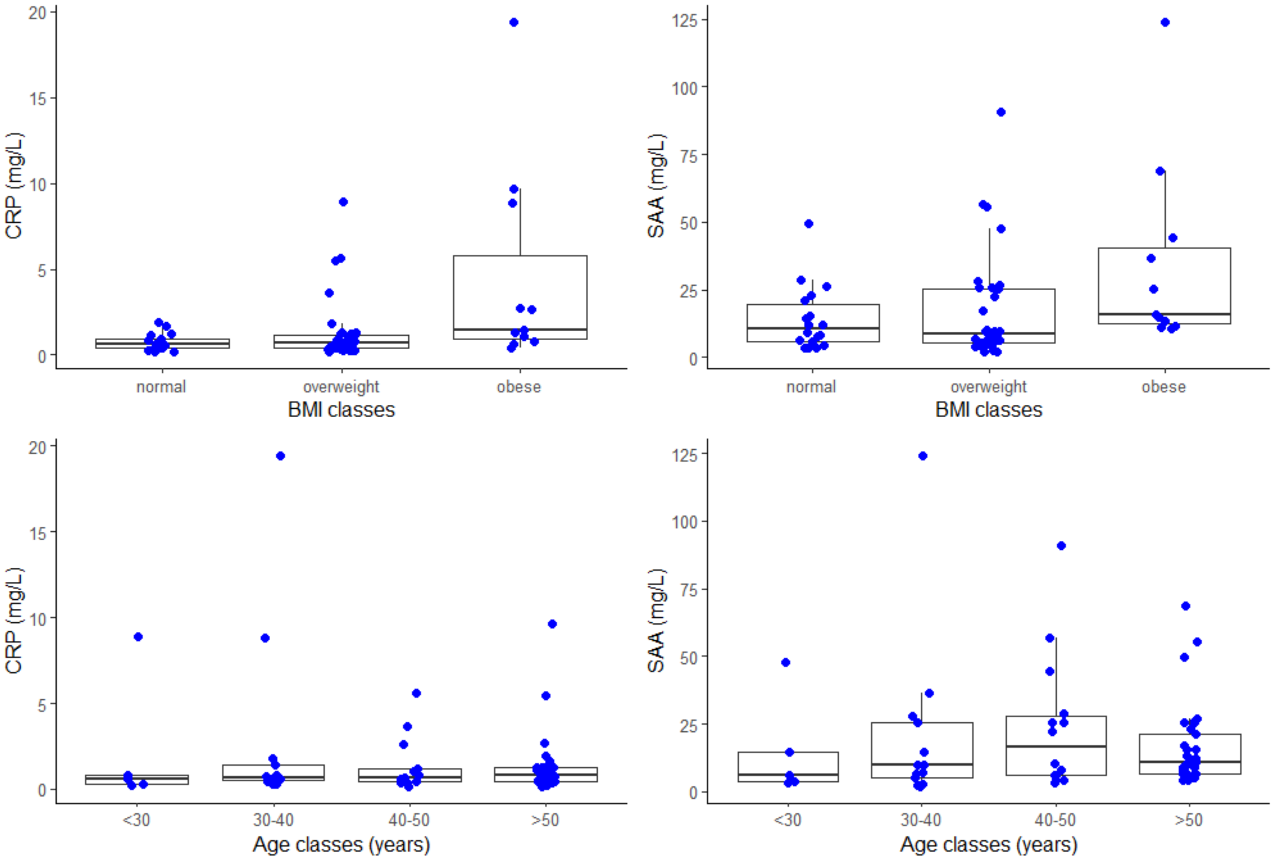

Figure S10 – Inflammation markers per classes of BMI and Age. BMI, body mass index (normal: 18.5-24.9; overweight: 25.0-29.9; obese: ≥30). CRP, C-reactive protein; SAA, serum amyloid A

References

1. Forchhammer L, Johansson C, Loft S, Moller L, Godschalk RW, Langie SA, Jones GD, Kwok RW, Collins AR, Azqueta A *et al*: **Variation in the measurement of DNA damage by comet assay measured by the ECVAG inter-laboratory validation trial**. *Mutagenesis* 2010, **25**(2):113-123.
